# Maternal smoking and smokeless tobacco use during pregnancy and offspring development: sibling analysis in an intergenerational Swedish cohort

**DOI:** 10.1093/ije/dyab095

**Published:** 2021-05-13

**Authors:** Paul Madley-Dowd, Michael Lundberg, Jon Heron, Stanley Zammit, Viktor H Ahlqvist, Cecilia Magnusson, Dheeraj Rai

**Affiliations:** 1 Centre for Academic Mental Health, Population Health Sciences, Bristol Medical School, University of Bristol, Bristol, UK; 2 Department of Global Public Health, Karolinska Institutet, Stockholm, Sweden; 3 MRC Centre for Neuropsychiatric Genetics and Genomics, Cardiff University, Cardiff, UK; 4 Centre for Epidemiology and Community Medicine, Region Stockholm, Stockholm, Sweden; 5 Avon and Wiltshire Partnership, NHS Mental Health Trust, Bristol, UK

**Keywords:** Intellectual disability, fetal growth restriction, maternal prenatal smoking, snus, siblings, confounding

## Abstract

**Background:**

The association between maternal smoking in pregnancy and offspring intellectual disability (ID) is less well understood than that of smoking and fetal growth restriction. As fetal growth and cognitive development may share similar confounding structures, comparison of the two associations may improve understanding of the causal nature of the association with ID. Furthermore, comparisons of smoking with smokeless tobacco use may aid identification of mechanisms of action.

**Methods:**

This was a cohort study of all Swedish births between 1999 and 2012 (*n* = 1 070 013), with prospectively recorded data. We assessed the association between maternal smoking during pregnancy and offspring outcomes ID and born small for gestational age (SGA). Analyses were repeated for snus use in pregnancy. Using a sibling design, we estimated within-family effects that control for shared sibling characteristics.

**Results:**

Those exposed to maternal smoking in pregnancy had increased odds of ID [odds ratio (OR) = 1.24, 95% confidence interval (CI): 1.16-1.33] and SGA (OR = 2.19, 95% CI: 2.11-2.27) after confounder adjustment. Within-family effects were found for SGA (OR = 1.44, 95% CI: 1.27-1.63) but not ID (OR = 0.92, 95% CI: 0.74-1.14). For snus use, the results for ID were similar to smoking. We found increased odds of offspring SGA among mothers who used snus in pregnancy in sensitivity analyses but not in primary analyses.

**Conclusions:**

Our findings are consistent with a causal effect of maternal smoking in pregnancy on risk of offspring born SGA but not on risk of ID. We found no evidence for a causal effect of snus use in pregnancy on ID and inconclusive evidence for SGA.

Key Messages

- Comparison of associations that are suspected to be the result of residual confounding with those that have a strong body of evidence for being causal can be useful for understanding the causal nature of the former association.
- In this study we used sibling designs to explore the association between maternal smoking in pregnancy and offspring risk of intellectual disability and compared this with analyses of the association between maternal smoking in pregnancy and offspring risk of being born small for gestational age (SGA).
- In models that hold fixed genetic and environmental factors shared between siblings, maternal smoking in pregnancy was associated with risk of being born SGA but not with risk of developing intellectual disability.
- We therefore provide evidence that is not consistent with a causal effect of maternal smoking in pregnancy on offspring intellectual disability.
- Snus use in pregnancy was not associated with either outcome in primary analyses. Sensitivity analyses did suggest an association between snus use in pregnancy and offspring SGA but we were unable to provide evidence of the causal nature of this association.

## Background

There has been much interest in the cognitive consequences of exposure to nicotine in pregnancy,^1^^,^^2^ due to its effects on fetal brain development processes, including neurogenesis, migration, differentiation and synaptogenesis.^3^ However, such associations may be attributable to confounding.

The association between nicotine exposure in pregnancy and extreme cognitive deficits such as intellectual disability (ID), defined as having an IQ of less than 70 alongside functional impairments,^4^ has been under-researched so far. Individuals with ID suffer from poor long-term outcomes and inequalities compared with the general population, such as increased mortality,^5^ socioeconomic disadvantage^6^^,^^7^ and worse access to and effectiveness of health care.^8–11^

A systematic review has suggested that smoking during pregnancy is associated with a small increase in the risk of offspring ID,^12^ though the studies included did not adequately account for confounding or information bias. Three better quality studies not included in the review found an association between smoking in pregnancy and offspring risk of ID, but each suggested that this may be the result of residual confounding.^13–15^ Further triangulation of evidence from different causal inference techniques is required to establish whether such an interpretation is likely.^16–18^ In contrast, the association between smoking in pregnancy and offspring fetal growth restriction has strong evidence of being causal in nature, from complementary causal inference designs.^19–21^ This latter association can be used as a positive control for smoking in pregnancy and offspring ID. By this we mean that, using the same causal inference methods, if an association is found for fetal growth restriction but not ID then this will support the interpretation that observational associations with ID are the result of residual confounding. If a causal effect of smoking in pregnancy on ID does exist, then a cross-context comparison between the associations of snus use and smoking in pregnancy with offspring ID can be used to investigate whether effects are the result of nicotine or of the combustible components of cigarette smoke. Snus is a moist, smokeless tobacco that is increasingly being used as a smoking cessation aid in Sweden,^22^^,^^23^ with some suggestion that it is more successful as an aid to stopping smoking than nicotine patches or gum.^24^^,^^25^ Snus delivers nicotine in quantities that are comparable to cigarette smoke though with slower absorption and higher plasma nicotine concentration over an extended period.^22^^,^^26^

Finally, the association between snus use in pregnancy and offspring fetal growth restriction is of public health relevance as it may guide whether snus may be a smoking alternative during pregnancy for those who have difficulties with cessation. Limited research has been performed on snus use in pregnancy, though evidence of associations with preterm delivery,^27^^,^^28^ offspring born small for gestational age (SGA)^29^ and stillbirth^30^^,^^31^ have been suggested. Research into snus use in pregnancy and offspring fetal growth restriction has provided mixed results. Two previous studies investigating snus use in pregnancy and risk of offspring born SGA provided no evidence for an association,^31^^,^^32^ and another found evidence of increased odds of SGA for those who used snus before and early into pregnancy relative to no use of tobacco products.^29^ None of these studies made use of causal inference methods and may be susceptible to the effects of unmeasured confounding.

The Swedish data registries provide the opportunity to use methods to better account for familial confounding structures. Sibling analyses are a powerful tool as they hold fixed shared genetic and environmental factors that can lead to residual confounding. In this project we aimed to: (i) use conventional analyses to investigate whether maternal smoking in pregnancy is associated with offspring risk of intellectual disability; (ii) use sibling analyses to investigate whether such associations can be accounted for by characteristics shared between siblings; (iii) use positive control and cross-context comparisons to learn more about the nature of the association; and (iv) investigate whether snus use in pregnancy influences fetal growth restriction.

## Methods

The study was approved by the Regional Ethical Review Board, Stockholm (Dnr: 2016/987–32). The requirement to obtain informed consent was waived by the Regional Ethical Review Board, Stockholm (Dnr: 2016/987–32). All research was performed in accordance with relevant guidelines/regulations.

### Cohort definition

**Figure 1:**
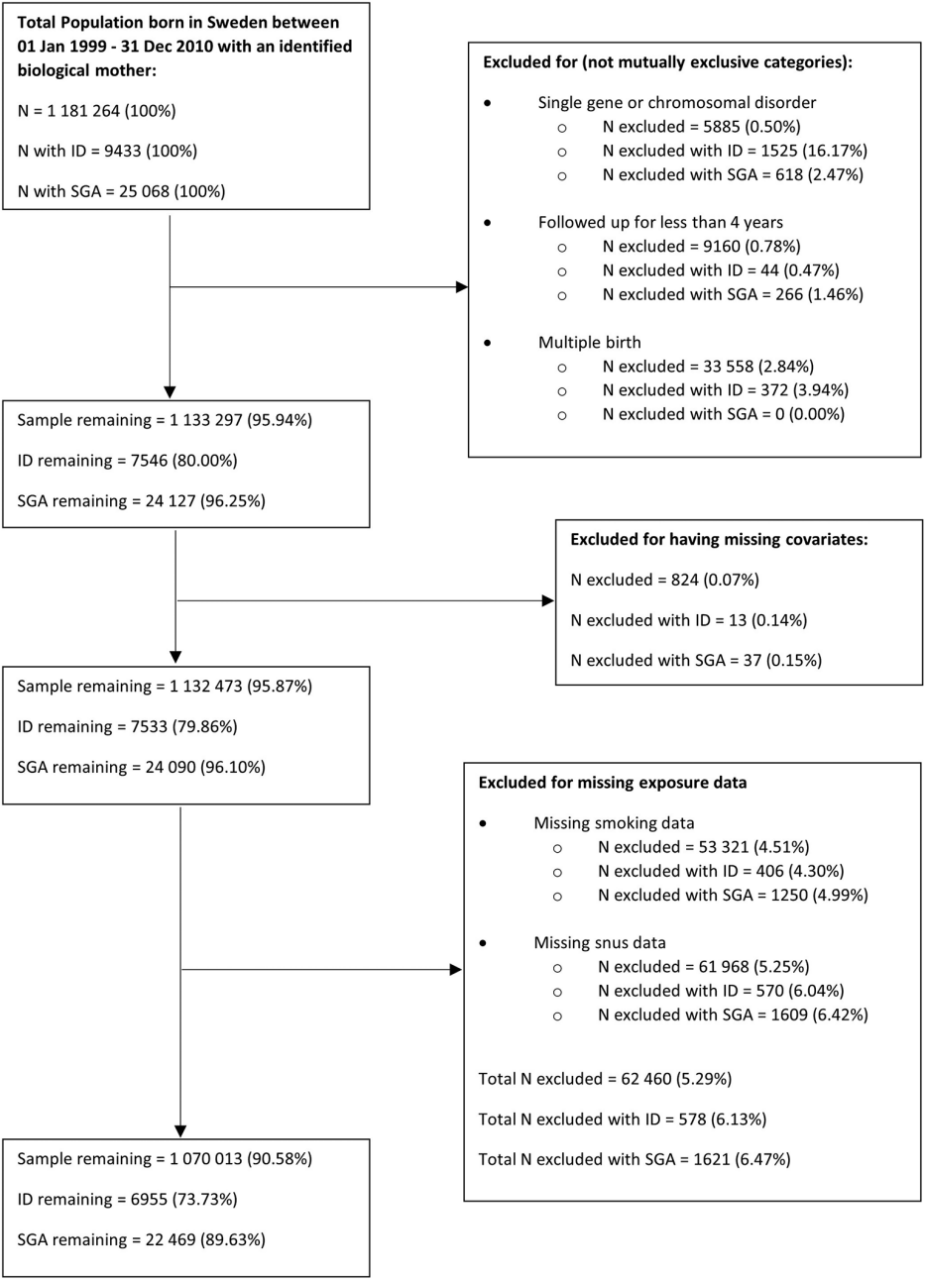
Flowchart of cohort derivation. Note: an additional 3280 individuals were excluded from small for gestational age (SGA) analyses due to missing SGA values. These were not removed from intellectual disability (ID) analyses as the SGA values may be missing due to the unobserved weight for gestational age value, which is on the hypothesized causal path from smoking to ID

The study cohort consisted of all individuals born in Sweden between 1 January 1999 and 31 December 2010 (*n* = 1 181 264; see Figure 1). Information contained in national registries was linked to cohort members and their parents. The registries included the Swedish Medical Birth Registry (MBR),^33^ the National Patient Registry (NPR)^34^ and the Swedish Longitudinal Integration Database for Health Insurance and Labour Market Studies (LISA).^35^ Data on maternal and paternal identity were obtained from the Swedish Multi-Generation Register (MGR).^36^

Most clinical contacts related to intellectual disability occur in an outpatient setting. The NPR started recording outpatient contact in addition to inpatient admissions in 2001.^34^ By defining the start year of the cohort as 1999, we were able to capture snus use in pregnancy from its earliest recording in the MBR while also capturing diagnoses from 2 years of age onwards for the oldest members in the cohort and from an earlier age for all other cohort members. We selected 2010 as the end year for inclusion in the cohort to allow a minimum of 4 years of follow-up until the end of 2014. The youngest and oldest members of the cohort were followed up until approximately 4 and 14 years of age, respectively.

### Exclusion criteria

Individuals were excluded from the cohort if they: had less than 4 years of follow-up (e.g. if the individual died before the age of 4 or had spent less than 4 years living in Sweden; *n* = 5885); had a genetic or chromosomal abnormality associated with ID that was identified using ICD-10 diagnoses^4^ in the NPR (specific diagnosis codes provided in section A1 of the Supplementary Methods, available as Supplementary data at *IJE* online; *n *= 9160); or were part of a multiple birth pregnancy (i.e. twins or triplets etc.; *n *= 33 558).

### Exposure definition: maternal smoking and snus use during pregnancy

Information about maternal smoking and snus use during pregnancy was obtained from the MBR for three time points: (i) 3 months preceding pregnancy; (ii) at the first antenatal contact (commonly around 10 weeks of pregnancy); and (iii) at 30–32 weeks of pregnancy. Further details of the method of collection of these data and their validity are provided in the Supplementary Methods, available as Supplementary data at *IJE* online (section A2).

Two binary variables were created, one for smoking and one for snus use, which indicated maternal use at any point during pregnancy (i.e. either at first antenatal contact or at 30–32 weeks). Categorical variables for the timing of exposure were created to indicate those whose mothers: (i) never smoked/used snus; (ii) used only before pregnancy; (iii) quit during pregnancy; or (iv) used throughout pregnancy. Individuals were excluded from analyses if they were missing the binary smoking (*n *= 53 321) or snus (*n* = 61 968) variables. The total number excluded for missing exposure data was 62 460.

### Outcome definitions

#### Intellectual disability (ID)

A binary indicator of ID was defined as having an ICD-10 code of F70-F79, recorded as a primary or secondary diagnosis in the NPR. Further details of diagnosis ascertainment and validity are contained in section A3 of Supplementary Methods, available as Supplementary data at *IJE* online.

#### Fetal growth restriction

Z-scores of birthweight for gestational age were obtained using the Swedish sex-specific reference curve for normal fetal growth.^37^ The MBR contains a binary indicator for being small for gestational age (SGA), defined as having a z-score value less than -2 (i.e. two standard deviations below the mean birthweight for a given gender and gestational age).

### Covariate and confounder definitions

The covariates and confounders adjusted for included child sex and parity, highest education level of either parent at the time of birth, quintiles of income adjusted for family size at the time of birth, any maternal or paternal psychiatric disorders before the birth of the child, and maternal country of origin and age at birth. Specific details of the confounder derivations are provided in section A4 of the Supplementary Methods, available as Supplementary data at *IJE* online. Individuals were excluded from analyses if they were missing data on any of the covariate or confounder variables (*n* = 824).

### Statistical analysis

#### Primary analyses

We repeated the following analyses for the outcomes ID and SGA and the exposures maternal smoking in pregnancy and maternal snus use in pregnancy. For each exposure-outcome combination we used logistic regression. To account for cohort effects of differing lengths of follow-up we adjusted for year of birth in all models that used an outcome of ID, even those referred to as unadjusted.

We ran four models for each exposure-outcome combination. Model 1 was unadjusted for any covariates. Model 2 adjusted for covariates and confounders. Model 3 adjusted for family-level smoking/snus use by including a term equal to the proportion of pregnancies in our cohort in which the mother was recorded as having smoked/used snus, thus making use of model formulation 2 suggested by Begg and Parides.^38^ Model 4 adjusted for all covariates, confounders and the family-level smoking/snus variable.

Adjustment for a family averaged exposure effect, as in model 3 and 4, allows the calculation of within-family (coefficient of the individual-level exposure) and between-family (coefficient of the family-level exposure) effects of smoking on child outcomes. The within-family effect is robust against confounders that are shared between the siblings. Failing to find a within-family effect after adjustment for the family-averaged exposure variable is consistent with familial confounding and there being no causal effect of the exposure on the outcome.^38^^,^^39^ Sjölander and colleagues detail the validity of within-between models for binary exposures,^40^ though they note that it is more accurate to describe the within-family effect as the causal effect within the exposure-discordant subpopulation. For ease of terminology we will continue to use the term ‘within-family effect’.

The family structure present within the cohort meant that the data violate the assumption of independence between observations which can lead to underestimation of standard errors. All models were therefore run using generalized estimating equations (GEE) with exchangeable correlation structures for family groups identified by having the same mother. This means that our analyses accounted for correlations between siblings. Full and half siblings were treated equivalently. Cousins and other relations were treated as independent.

#### Secondary analyses

We assessed whether timing of exposure was associated with our outcomes. We used four models to assess the association and repeated these analyses for the outcomes SGA and ID. Models 1 and 2, performed unadjusted and adjusted for confounders, respectively, were logistic regressions using GEE of the outcome on the categorical timing exposure. Models 3 and 4, again performed unadjusted and adjusted for confounders, respectively, were conditional logistic regressions of the outcome on the categorical timing exposure, conditional on family grouping. Models 1 and 2 therefore provide population-averaged estimates, and Models 3 and 4 provide within-family estimates.

#### Sensitivity analyses

We performed three sensitivity analyses, described in detail in section A5 of the Supplementary Methods, available as Supplementary data at *IJE* online. Briefly, we examined: (i) whether inclusion of snus users in the non-smoking comparison group (and smokers in the non-snus user comparison group) influenced our results; (ii) whether potentially increased measurement error in the exposure variable among exposure-discordant siblings influenced our conclusions; and (iii) whether the pattern of change of smoking across pregnancies (i.e. starting vs stopping smoking) influenced effect estimates, thereby indicating the presence of carry-over effects.^41^

## Results

### Description of the cohort

Descriptive statistics of the cohort, separated by smoking status and by snus use, are presented in Table 1. Maternal smoking in pregnancy was more prevalent in the cohort than snus use in pregnancy (8.78% vs 1.37%). Both smoking and snus use were socially patterned; however, the strength of that social patterning was greater for smokers. Smokers were more likely to have low- or mid-level education and low income, whereas snus users were more likely to have mid-level education and mid-level income. Both smokers and snus users were more likely to have a psychiatric disorder diagnosis before the birth of their child. Smokers [mean: 28.8 years; standard deviation (SD): 5.95] were younger on average than non-smokers (mean: 30.7 years; SD: 5.00). In comparison, the average age of snus users (mean: 30.4 years; SD: 5.51) was closer to that of non-users (mean: 30.6 years; SD: 5.11). Further descriptive results separated by categories of family-level smoking/snus use, by timing of smoking/snus use and by patterns of change in smoking status across pregnancy, can be viewed in the Supplementary Results, section B1, available as Supplementary data at *IJE* online.

**Table 1:** Cohort characteristics by exposure status during pregnancy

		*n* (%)			
Variable	Level	Non-smokers	Smokers	Non-snus users	Snus users
Total		976 035 (100.00)	93 978 (100.00)	1 055 348 (100.00)	14 665 (100.00)
Intellectual disability	No	970 134 (99.40)	92 924 (98.88)	1 048 519 (99.35)	14 539 (99.14)
	Yes	5901 (0.60)	1054 (1.12)	6829 (0.65)	126 (0.86)
Small for gestational age	No	954 572 (97.80)	89 692 (95.44)	1 029 957 (97.59)	14 307 (97.56)
	Yes	18 504 (1.90)	3965 (4.22)	22 152 (2.10)	317 (2.16)
Sex	Female	474 465 (48.61)	45 469 (48.38)	512 840 (48.59)	7094 (48.37)
	Male	501 570 (51.39)	48 509 (51.62)	542 508 (51.41)	7571 (51.63)
Parity	1	435 909 (44.66)	40 054 (42.62)	469 671 (44.50)	6292 (42.90)
	2	362 557 (37.15)	28 990 (30.85)	386 451 (36.62)	5096 (34.75)
	3 or more	177 569 (18.19)	24 934 (26.53)	199 226 (18.88)	3277 (22.35)
Highest parental education	Pre-age 16	35 861 (3.67)	13 876 (14.77)	49 097 (4.65)	640 (4.36)
	Age 16-18	378 936 (38.82)	63 647 (67.73)	434 338 (41.16)	8245 (56.22)
	Post-age 18	561 238 (57.50)	16 455 (17.51)	571 913 (54.19)	5780 (39.41)
Adjusted family income	1	102 100 (10.46)	18 644 (19.84)	119 319 (11.31)	1425 (9.72)
	2	193 375 (19.81)	32 940 (35.05)	222 371 (21.07)	3944 (26.89)
	3	220 396 (22.58)	21 861 (23.26)	238 255 (22.58)	4002 (27.29)
	4	231 394 (23.71)	14 066 (14.97)	242 298 (22.96)	3162 (21.56)
	5	228 770 (23.44)	6467 (6.88)	233 105 (22.09)	2132 (14.54)
Maternal anxiety diagnosis	No	949 353 (97.27)	86 679 (92.23)	1022 267 (96.87)	13 765 (93.86)
	Yes	26 682 (2.73)	7299 (7.77)	33081 (3.13)	900 (6.14)
Maternal depression diagnosis	No	957 545 (98.11)	89 152 (94.86)	1 032 714 (97.86)	13 983 (95.35)
	Yes	18 490 (1.89)	4826 (5.14)	22 634 (2.14)	682 (4.65)
Maternal psychosis diagnosis	No	973 070 (99.70)	93 089 (99.05)	1 051 612 (99.65)	14 547 (99.20)
	Yes	2965 (0.30)	889 (0.95)	3736 (0.35)	118 (0.80)
Maternal addiction diagnosis	No	965 089 (98.88)	87 978 (93.62)	1 039 026 (98.45)	14 041 (95.74)
	Yes	10 946 (1.12)	6000 (6.38)	16 322 (1.55)	624 (4.26)
Any maternal psychiatric diagnosis	No	929 617 (95.24)	80 419 (85.57)	997 001 (94.47)	13 035 (88.89)
	Yes	46 418 (4.76)	13 559 (14.43)	58 347 (5.53)	1630 (11.11)
Any paternal psychiatric diagnosis	No	942 241 (96.54)	84 672 (90.10)	1 013 191 (96.01)	13 722 (93.57)
	Yes	33 794 (3.46)	9306 (9.90)	42 157 (3.99)	943 (6.43)
Any maternal neurodevelopmental diagnosis	No	974 771 (99.87)	93 216 (99.19)	1 053 411 (99.82)	14 576 (99.39)
	Yes	1264 (0.13)	762 (0.81)	1937 (0.18)	89 (0.61)
Any paternal neurodevelopmental diagnosis	No	974 536 (99.85)	93 220 (99.19)	1 053 143 (99.79)	14 613 (99.65)
	Yes	1499 (0.15)	758 (0.81)	2205 (0.21)	52 (0.35)
Maternal country of origin	Africa	29 178 (2.99)	915 (0.97)	29 985 (2.84)	108 (0.74)
	Americas	10 512 (1.08)	658 (0.70)	11 110 (1.05)	60 (0.41)
	Asia	30 865 (3.16)	1469 (1.56)	32 116 (3.04)	218 (1.49)
	Europe	47 317 (4.85)	7651 (8.14)	54 763 (5.19)	205 (1.40)
	Middle East	54 858 (5.62)	4531 (4.82)	59 197 (5.61)	192 (1.31)
	Oceania	422 (0.04)	26 (0.03)	443 (0.04)	5 (0.03)
	Scandinavia	15 274 (1.56)	2224 (2.37)	17 287 (1.64)	211 (1.44)
	Swedish	787 609 (80.69)	76 504 (81.41)	850 447 (80.58)	13 666 (93.19)
Birth year	1999–2001	206 348 (21.14)	20 786 (22.12)	224 778 (21.30)	2356 (16.07)
	2002–04	238 757 (24.46)	28 067 (29.87)	262 758 (24.90)	4066 (27.73)
	2005–07	248 194 (25.43)	22 508 (23.95)	266 973 (25.30)	3729 (25.43)
	2008–10	282 736 (28.97)	22 617 (24.07)	300 839 (28.51)	4514 (30.78)
Any maternal smoking in pregnancy	No			962 734 (91.22)	13 301 (90.70)
	Yes			92 614 (8.78)	1364 (9.30)
Any maternal snus use in pregnancy	No	962 734 (98.64)	92 614 (98.55)		
	Yes	13 301 (1.36)	1364 (1.45)		

### Missing data assessment

Full details are presented in the Supplementary Results, section B2, available as Supplementary data at *IJE* online. Briefly, missing data in covariates were socially patterned with those with lower income and education, and those born in later cohort years more likely to be excluded for having a missing covariate. Missing data in the exposure was more likely in those with lower income and less likely for those born in later cohort years. Smokers were more likely to have been excluded for having missing snus data and vice versa. Both diagnosis of ID and being born SGA were associated with increased risk of exclusion for missing covariate and exposure data.

### Primary analyses

Table 2 shows the results for the primary analyses using offspring ID as an outcome. Conventional analyses (i.e. Models 1 and 2) showed that both smoking and snus use in pregnancy were associated with increased odds of ID following adjustment for confounders. When separated into within-family and between-family effects, there was evidence of between-family but not within-family effects of smoking and snus use in pregnancy, before and after adjustment for confounders.

**Table 2:** Primary analysis of the association between exposure and offspring intellectual disability (ID)

		Smoking in pregnancy		Snus use in pregnancy	
Model	Coefficient	OR^a^	95% CI	OR^a^	95% CI
1. Conventional unadjusted^b^	Population-averaged	1.80	(1.68-1.93)	1.36	(1.14-1.63)
2. Conventional adjusted^c^	Population-averaged	1.24	(1.16-1.33)	1.28	(1.06-1.52)
3. Within-between unadjusted^b^^,d^	Within-family	0.91	(0.73-1.14)	0.88	(0.60-1.29)
	Between-family	2.13	(1.70-2.68)	1.74	(1.14-2.67)
4. Within-between adjusted^c^^,d^	Within-family	0.92	(0.74-1.14)	0.87	(0.59-1.28)
	Between-family	1.40	(1.12-1.76)	1.61	(1.05-2.48)

a Estimates produced using a total sample size of 1 070 013 individuals from 703 835 families including 6955 cases of ID.

b Model adjusted for year of birth.

c Model adjusted for year of birth, sex, parity, highest parental education, income, parental psychiatric history, maternal country of origin and maternal age at birth.

d Model adjusted for family-averaged exposure.

The results of our primary analyses using offspring SGA as the outcome are presented in Table 3. Smoking in pregnancy was associated with a population-averaged increased odds of offspring SGA after adjustment for confounders. Models 3 and 4 showed that smoking in pregnancy was associated with increased odds of SGA for both the within-family and between-family effects. Snus use in pregnancy was not associated with offspring SGA in any model. For most models, the confidence intervals for estimates of the effect of snus use were not compatible with that of smoking, suggesting that the absence of an association between snus use and SGA was not the result of a lack of power for a rarer exposure.

**Table 3:** Primary analysis of the association between exposure and offspring born small for gestational age (SGA)

		Smoking in pregnancy		Snus use in pregnancy	
Model	Coefficient	OR^a^	95% CI	OR^a^	95% CI
1. Conventional unadjusted	Population-averaged	2.26	(2.18-2.35)	1.02	(0.92-1.15)
2. Conventional adjusted^b^	Population-averaged	2.19	(2.11-2.27)	1.05	(0.94-1.17)
3. Within-between unadjusted^c^	Within-family	1.68	(1.50-1.89)	1.01	(0.80-1.26)
	Between-family	1.40	(1.23-1.58)	1.02	(0.79-1.33)
4. Within-between adjusted^b^^,c^	Within-family	1.44	(1.27-1.63)	1.07	(0.84-1.36)
	Between-family	1.60	(1.41-1.83)	0.98	(0.75-1.29)

a Estimates produced using a total sample size of 1 066 733 individuals from 702 475 families including 22 469 cases of SGA.

b Model adjusted for year of birth, sex, parity, highest parental education, income, parental psychiatric history, maternal country of origin and maternal age at birth.

c Model adjusted for family-averaged exposure.

### Secondary analyses

The results for offspring ID are presented in Table 4. An exposure duration response of increased odds of ID was found for smoking and using snus later into pregnancy in conventional models only (Models 1 and 2). In conditional logistic models that calculated within-family estimates of the exposure-ID association, no association was found for smoking for any exposure timing. For smoking (Model 2) and snus use (Model 1) there was evidence of reduced odds of ID among those who quit smoking before pregnancy. Within-family estimates (Models 3 and 4) of the snus use-ID association showed evidence of decreased odds of ID among those who quit using snus during pregnancy compared with those who did not use snus at any time.

**Table 4:** Secondary analysis of the association between exposure timing and offspring intellectual disability (ID)

		Smoking in pregnancy		Snus use in pregnancy	
Model	Coefficient	OR^a^	95% CI	OR^b^	95% CI
1. Conventional unadjusted^c^ (population-averaged estimates)	Non-user	1.00		1.00	
	User before pregnancy only	1.04	(0.96-1.13)	0.76	(0.60-0.97)
	Quit during pregnancy	1.40	(1.19-1.64)	1.07	(0.76-1.50)
	Used late into pregnancy	1.79	(1.64-1.95)	2.02	(1.49-2.75)
2. Conventional adjusted^c^^,d^ (population-averaged estimates)	Non-user	1.00		1.00	
	User before pregnancy only	0.90	(0.82-0.98)	0.85	(0.67-1.08)
	Quit during pregnancy	1.04	(0.88-1.22)	1.04	(0.74-1.47)
	Used late into pregnancy	1.17	(1.07-1.28)	1.80	(1.32-2.46)
3. Unadjusted conditional logistic^c^ (within-family estimates)	Non-user	1.00		1.00	
	User before pregnancy only	0.94	(0.76-1.16)	0.84	(0.52-1.37)
	Quit during pregnancy	0.96	(0.65-1.41)	0.43	(0.19-0.97)
	Used late into pregnancy	1.06	(0.75-1.50)	1.06	(0.44-2.54)
4. Adjusted conditional logistic^c^^,d^ (within-family estimates)	Non-user	1.00		1.00	
	User before pregnancy only	0.91	(0.73-1.13)	0.87	(0.53-1.41)
	Quit during pregnancy	0.94	(0.63-1.40)	0.41	(0.18-0.94)
	Used late into pregnancy	1.00	(0.70-1.43)	0.98	(0.41-2.36)

a Estimates conventional models produced using a total sample size of 1 050 528 individuals from 694 227 families including 6658 cases of ID. Estimates for conditional logistic models produced using a total sample size of 8412 individuals including 3612 cases of ID.

b Estimates for conventional models produced using a total sample size of 1 063 701 individuals from 700 988 families including 6905 cases of ID. Estimates for conditional logistic models produced using a total sample size of 8791 individuals including 3762 cases of ID.

c Model adjusted for year of birth.

d Model adjusted for year of birth, sex, parity, highest parental education, income, parental psychiatric history, maternal country of origin and maternal age at birth.

Table 5 shows the results of our timing analyses for offspring SGA as the outcome. Smoking longer into pregnancy was associated with a duration-responsive increase in odds of offspring SGA in conventional and conditional logistic analyses (Models 1–4). For smoking (Model 2) and snus use (Models 1 and 2) in pregnancy, there was evidence for a reduced risk of offspring SGA in conventional models for mothers who gave up using before pregnancy compared with those who did not use snus at any time. There was no other evidence for an association between snus use and offspring SGA.

**Table 5:** Secondary analysis of the association between exposure timing and offspring born small for gestational age (SGA)

		Smoking in pregnancy		Snus use in pregnancy	
Model	Coefficient	OR^a^	95% CI	OR^b^	95% CI
1. Conventional unadjusted (population-averaged estimates)	Non-user	1.00		1.00	
	User before pregnancy only	1.04	(1.00-1.09)	0.82	(0.73-0.92)
	Quit during pregnancy	1.46	(1.34-1.60)	0.90	(0.74-1.10)
	Used late into pregnancy	2.43	(2.32-2.53)	0.89	(0.70-1.15)
2. Conventional adjusted^c^ (population-averaged estimates)	Non-user	1.00		1.00	
	User before pregnancy only	0.90	(0.86-0.94)	0.78	(0.70-0.88)
	Quit during pregnancy	1.30	(1.19-1.42)	0.91	(0.75-1.12)
	Used late into pregnancy	2.37	(2.26-2.49)	0.96	(0.74-1.23)
3. Unadjusted conditional logistic (within-family estimates)	Non-user	1.00		1.00	
	User before pregnancy only	1.77	(1.55-2.03)	1.16	(0.86-1.58)
	Quit during pregnancy	2.03	(1.58-2.60)	1.13	(0.72-1.77)
	Used late into pregnancy	2.87	(2.30-3.58)	0.96	(0.47-1.96)
4. Adjusted conditional logistic^c^ (within-family estimates)	Non-user	1.00		1.00	
	User before pregnancy only	0.97	(0.83-1.12)	0.92	(0.66-1.28)
	Quit during pregnancy	1.14	(0.87-1.49)	1.37	(0.83-2.25)
	Used late into pregnancy	1.79	(1.42-2.27)	1.39	(0.65-2.97)

a Estimates for conventional models produced using a total sample size of 1 047 385 individuals from 692 911 families including 21 522 cases of SGA. Estimates for conditional logistic models produced using a total sample size of 21 943 individuals including 9985 cases of SGA.

b Estimates for conventional models produced using a total sample size of 1 060 437 individuals from 699 629 families including 22 310 cases of SGA. Estimates for conditional logistic models produced using a total sample size of 22 899 individuals including 10 402 cases of SGA.

c Model adjusted for year of birth, sex, parity, highest parental education, income, parental psychiatric history, maternal country of origin and maternal age at birth.

### Sensitivity analyses

The results of the sensitivity analyses are presented and described in detail in section B3 of the Supplementary Results, available as Supplementary data at *IJE* online. The repetition of analyses using a cleaner comparison did not materially change the estimates or conclusions of analyses for smoking-ID associations, smoking-SGA associations or snus-ID associations. Using the new comparison group, unadjusted and adjusted conventional models showed slightly increased odds of SGA among mothers who used snus during pregnancy. It is unclear from within-family and between-family effect estimates whether these associations were the result of causal effects or residual confounding. The second set of analyses suggested that our conclusions would not be substantially changed as a result of increased exposure misclassification among exposure-discordant families. The third set of analyses showed that we were able to rule out the influence of carry-over effects for the outcome ID but not for SGA.

## Discussion

We have found evidence that supports a causal role of smoking in pregnancy on offspring fetal growth restriction, as measured by SGA, and no evidence of a causal influence of smoking or snus use in pregnancy on risk of offspring ID. Both population-averaged and within-family effect estimates suggested a role of smoking on fetal growth restriction. The within-family effect can be interpreted as meaning that individual-level exposure to maternal smoking in pregnancy, holding fixed shared familial genetics and environment, is associated with being born SGA. Maternal smoking and snus use in pregnancy were both associated with increased population-averaged odds of ID. In both cases this was shown to be driven by the between-family effect and not the within-family effect; a finding that is not consistent with a causal effect. Our primary and sensitivity analyses provided conflicting evidence for the causal nature of the association between snus use in pregnancy and offspring fetal growth restriction. In primary analyses no association was found between snus use in pregnancy and offspring fetal growth restriction, even in unadjusted conventional models. In sensitivity analyses a population-averaged effect was suggested, but there was insufficient precision in sibling analyses to suggest whether this was the result of within-family or between-family effects.

The results of our timing analyses supported our conclusions of the causal nature of each exposure-outcome association. A duration response was found for the within-family effect estimates of smoking in pregnancy and offspring SGA but not for any other investigated association. We did, however, obtain some unusual results for those who quit smoking or snus use. Compared with no use, giving up smoking or snus before pregnancy was associated with reduced odds of SGA and ID in some conventional models. Giving up snus during pregnancy was also associated with reduced odds of ID, using within-family estimates. Although a protective effect of nicotine at critical time points may be possible, we believe that these results could potentially be explained by the characteristics of mothers who are able to quit using an addictive substance at an important time in order to benefit their child’s health.

Our study strengthens the current body of evidence for a causal effect of maternal smoking in pregnancy on fetal growth restriction and provides strong support to the suggestion that the association between smoking in pregnancy and offspring ID is the result of residual confounding.^13–15^ To our knowledge, no previous research has investigated the association between prenatal exposure to snus and offspring ID.

Our study used a similar Swedish cohort and an identical definition of SGA to those in the studies by Baba *et al*.,^29^ who found an association between maternal snus use in pregnancy and offspring born SGA, and to those of Wikström *et al.*^31^ who did not. Our primary analyses did not suggest any association between snus use in pregnancy and offspring risk of SGA, though our sensitivity analyses, using a potentially cleaner comparison group, suggested exposure was associated with increased risk. The conflicting results and insufficient precision of the within- and between-family effect estimates in the sensitivity analysis mean that further investigation is required to determine if snus use during pregnancy causally influences the risk of offspring born SGA.

### Strengths and limitations

Our study suffers from limitations that all registry-based studies are subject to, including potential misclassification, missing data and residual confounding. A previous study has shown good validity of measures of smoking during pregnancy in the MBR,^42^ though we note that no such checks of validity have been performed for snus use during pregnancy. Sensitivity analysis (ii) suggested that our results would not be substantially changed if there were high levels of exposure misclassification in the exposure-discordant group, though we only tested one scenario of exposure misclassification.

We have used complete case analysis only, which may be biased for logistic regression when the missing data are related to both the exposure and the outcome,^43^ as is the case for our dataset. Previous work has shown that there is smaller bias at smaller proportions of missing data.^44^ The small quantity of missing data in our dataset may limit the bias; however, the strong associations between smoking/snus use status and missing data for snus use/smoking data may have led to greater quantities of bias. Given that smokers/snus users and those with ID were both more likely to have been excluded for missing data, this may have biased the association towards the null.

Our study attempted to account for residual confounding by using a sibling design which holds fixed shared familial genetics and environment. We were unable to easily control for the non-shared confounders of siblings, which have been shown to bias the results of sibling designs,^45^ due to the varying size of families. The antenatal nature of the associations that we are investigating, however, mean that the non-shared confounders will be limited to changes in environment between pregnancies, as the mother’s genetics will not change. Sibling designs are also subject to bias from carry-over effects, for example where the outcome of the first sibling influences the exposure of the second sibling. Our sensitivity analysis ruled out carry-over effects for ID but was not able to do so for SGA. The bias arising from such a pattern has been shown to be difficult to quantify.^41^

A key strength of our study is the relationship between the associations investigated. There is strong evidence for a causal effect of smoking in pregnancy on fetal growth restriction, whereas the causal nature of the association between smoking in pregnancy and ID is unclear. We found evidence using within-between models of a causal influence of smoking in pregnancy on fetal growth restriction but not ID. As this is in line with previous findings, the former association can be thought of as a positive control of the latter association, thereby strengthening our confidence in the evidence produced for the non-causal influence of exposure to maternal smoking in pregnancy on offspring ID. Further, had a causal effect been suggested, comparison of the influence of smoking and snus use in pregnancy on offspring ID would have provided a cross-context comparison which could have been useful for identifying whether nicotine or combustible components of smoking were involved in biological mechanisms.

In conclusion, our study has provided no evidence for a causal effect of smoking or snus use in pregnancy on risk of offspring ID, instead suggesting that associations are the result of residual confounding. We found conflicting evidence regarding whether snus use in pregnancy is associated with the risk of offspring being born SGA. Neither finding suggests that smoking or snus use in pregnancy is safe. Smoking in pregnancy has well established negative effects on offspring health, whereas research into snus use in pregnancy is in its infancy. Further assessments of the health costs and benefits of snus use in pregnancy relative to smoking need to be performed before guidance can be given regarding whether snus use is a suitable alternative to smoking or even whether it should be used as a cessation aid during this period.

## Supplementary data

Supplementary data are available at *IJE* online.

## Funding

This research was funded by the Wellcome Trust [Grant ref: 203776/Z/16/A]. For the purpose of Open Access, the author has applied a CC BY public copyright licence to any Author Accepted Manuscript version arising from this submission. S.Z. and D.R. were supported by the NIHR Biomedical Research Centre at University Hospitals Bristol NHS Foundation Trust and the University of Bristol (Grant ref: BRC-1215–2011). The views expressed in this publication are those of the author(s) and not necessarily those of the NHS, the National Institute for Health Research or the Department of Health and Social Care. D.R., M.L., V.A. and C.M. were supported by a National Institutes of Health (Grant ref: 1R01NS107607-01A1). The views expressed in this publication are those of the author(s) and not necessarily those of the National Institutes of Health.

## Data availability

The data underlying this article cannot be shared publicly for ethical reasons.

## Supplementary Material

dyab095_Supplementary_Data
