# Supplementary material for: Maternal smoking and smokeless tobacco use during pregnancy and offspring development: sibling analysis in an intergenerational Swedish cohort
Source: Int J Epidemiol. 2021 May 13;50(6):1840–51. doi: 10.1093/ije/dyab095 (PMC8743113; doi:10.1093/ije/dyab095)
Supplement: dyab095_Supplementary_Data [file dyab095_supplementary_data.pdf]

Supplementary material

Maternal smoking and snus use during pregnancy and offspring development: sibling analysis in an intergenerational Swedish cohort

Contents

A - Supplementary Methods 3
A1 - Exclusion criteria 3
A2 - Further exposure information 4
A3 - Further information on diagnoses of intellectual disability 5
A4 - Confounder definitions 6
A4.1 - Parity 6
A4.2 - Parental education 6
A4.3 - Parental income 6
A4.4 - Parental psychiatric disorders 6
A4.5 - Maternal country of origin 7
A4.6 - Parental age at birth 7
A5 - Sensitivity analyses 8
A5.1 - Sensitivity analysis (i) – influence of using a cleaner comparison group 8
A5.2 - Sensitivity analysis (ii) – influence of measurement error in the exposure variable among exposure discordant families 8
A5.3 - Sensitivity analysis (iii) – test of whether the pattern of smoking change across pregnancies influences the within-family estimate (ruling out carry-over effects) 8
B - Supplementary Results 10
B1 - Cohort descriptives 10
B1.1 - Descriptives separated by categories of family level smoking 10
B1.2 - Descriptives separated by timing of smoking exposure 13
B1.3 - Descriptives separated by timing of snus exposure 16
B1.4 - Descriptives of cohort for sensitivity analysis (ii) separated by change in smoking behaviour across first two pregnancies 19
B2 - Missing data 21
B2.1 - Descriptives for missing covariate data 21
B2.2 - Descriptives for missing exposure data 23
B3 - Sensitivity analyses 25
B3.1 - Sensitivity analysis (i) – influence of using a cleaner comparison group 25

B3.2 - Sensitivity analysis (ii) – influence of measurement error in the exposure variable among exposure discordant families 25

B3.3 - Sensitivity analysis (iii) – test of whether the pattern of smoking change across pregnancies influences the within-family estimate (ruling out carry-over effects) 26

References 32

A - Supplementary Methods

A1 - Exclusion criteria

Table A1.1 - ICD-10 diagnosis codes for genetic and chromosomal abnormalities associated with intellectual disability that were used in exclusion criteria.

ICD-10 Code	Description
Q89.8	Williams syndrome
Q87.1	Prader-Willi syndrome
Q87.2	Rubinstein-Taybi syndrome
Q44.7	Alagille syndrome
D82.1	DiGeorge syndrome
Q85.0	Neurofibromatosis (non-malignant)
Q85.1	Tuberous sclerosis
Q90-Q99	Chromosomal abnormalities, not elsewhere specified
E70-E72	Metabolic disorders

A2 - Further exposure information

In Sweden, mothers are interviewed by trained midwives using a standardised questionnaire that records current smoking (non-smoking, 1-9 cigarettes/day or more than 9 cigarettes per day) at two separate visits during pregnancy (1, 2). The first antenatal visit where data are recorded usually occurs between 8-12 weeks gestation and the second is between 30-32 weeks. Smoking at 3 months prior to pregnancy is recorded at first visit on the same scale as current smoking.

Data on maternal smoking during pregnancy in the Medical Birth Register (MBR) has been validated against cotinine measures in maternal and umbilical cord blood (1). There was high agreement between MBR data on smoking early in pregnancy and maternal cotinine measures at the time of birth. 95% of non-smokers were classed as non-smokers using cotinine measurement. 87% of those recorded as smokers in the MBR were smokers according to cotinine measurement at birth. To date, the data in the MBR have not been validated for snus use but considering snus use is widespread and socially acceptable in Sweden, and there is more social pressure to misreport smoking, we do not think this variable is likely to have major reporting bias.

A3 - Further information on diagnoses of intellectual disability

All children in Sweden are regularly assessed at clinics using medical and developmental screening tools. A mandatory assessment of motor, language, cognitive, and social development is conducted at age 4 years. Children who are suspected of having a developmental disorder are referred to a specialist team for further assessment with any diagnostic information reported to the NPR (3, 4). As a result, the NPR should systematically ascertain diagnoses of ID.

Validation of the national patient registry (NPR) shows that coverage of hospital based outpatient care is around 80% (near 100% for public care givers but missing entirely from private care givers) (5). Validity of diagnoses for ID in the NPR have been undertaken in the context of autism spectrum disorders (ASD) but not with specific focus on ID itself (6); 88.5% of ASD without ID cases were confirmed and 75.6% of ASD with ID cases were confirmed. Studies of the validity of other neurological conditions (OCD and tick disorders) have shown high positive predictive values (>90%) using ICD-10 codes in the NPR (7).

A4 - Confounder definitions

The confounders included in adjusted models were selected based on prior literature or clinical knowledge of plausible associations with both maternal smoking in pregnancy and intellectual disability or offspring risk of being born SGA. The selected variables for adjustment in statistical models were child sex and parity, highest education level of either parent at the time of birth, quintiles of income adjusted for family size at the time of birth, any maternal or paternal psychiatric disorders before the birth of the child and maternal country of origin and age at birth.

A4.1 - Parity

Parity was grouped into a categorical variable with 3 levels: 1, 2 and 3+.

A4.2 - Parental education

Parental educational attainment was obtained from the Swedish LISA (Longitudinal integration database for health insurance and labour market studies) database (8). Education was grouped into 3 levels: High school (pre-age 16 education); Gymnasium (age 16-18, equivalent to A levels in the UK and 11th-12th Grade in the USA); University level (post-age 18 education). The highest level of either parent at the time of the child's birth was used.

A4.3 - Parental income

Household income in the year of the child's birth was obtained from the Swedish LISA database (8). The value was adjusted for family size and placed into quintiles for each year in order to account for inflation.

A4.4 - Parental psychiatric disorders

Indicator variables were derived for diagnoses of anxiety disorders, depressive disorders, psychotic disorders and substance use disorders (excluding nicotine related disorders) in the National Patient Register (NPR) (5) at any time before the child's birth; see Table A4.1 for the list of ICD-9 and ICD-10 codes used to define these disorders. Due to the low prevalence of each disorder in the cohort (see Table 1 of the main text) we combined the indicator variables into a single variable of any psychiatric disorder at any time before the child's birth.

Table A4.1: ICD-9 and ICD-10 codes used to define parental psychiatric history.

Disorder	ICD-9 codes	ICD-10 codes
Anxiety disorders	300.0, 300.2, 300.3, 308, 309	F40-F43
Depressive disorders	296.1, 298.0, 300.4, 311	F32-F39
Psychotic disorders (affective and non-affective)	295, 296.0, 296.2, 296.3, 296.4, 297, 298.2, 298.3, 298.4, 298.8, 298.9	F20-F29, F30-F31
Substance use disorders	291.0-291.9<br>303.0-303.9<br>304.0-304.9<br>305.0-305.9 (*minus 305.1*)	F10-F16, F18-F19

A4.5 - Maternal country of origin

Maternal country of origin was be obtained using the Multi-Generational Register (MGR) (9). The variable was categorised into the following levels: Sweden; Scandinavia (Denmark and Finland); Europe; Middle East; Americas (North and South America); Asia; Africa; Oceania.

A4.6 - Parental age at birth

Parental age at the time of the child's birth was derived from the parent and child's date of birth and included as a continuous variable.

A5 - Sensitivity analyses

A5.1 - Sensitivity analysis (i) – influence of using a cleaner comparison group

Inclusion of snus-users in the comparison group for smoking analyses (i.e. non-smoking mothers) could lead to a violation of the consistency assumption as some of those who were not exposed to smoking during pregnancy would still be exposed to nicotine. The same can be said for the inclusion of smokers in the comparison group for snus analyses (i.e. non-snus using mothers). To test whether this substantially affected the results of analyses we repeated the primary analyses excluding all those in the non-smoking/non-snus using comparison group who used snus/smoked in pregnancy. Family averaged exposure was recalculated after the exclusions. The secondary analyses of exposure timing were also repeated with those who used snus at any time excluded from smoking timing analyses and those who smoked at any time excluded from snus use timing analyses.

A5.2 - Sensitivity analysis (ii) – influence of measurement error in the exposure variable among exposure discordant families

An assessment of potential biases in sibling designs highlighted that the exposure discordant group, which drives the within-family estimate, is more likely to contain exposure misclassification than the population as a whole (10). This is because mothers are likely to behave similarly across pregnancies (see counts in section B1.1 of Appendix B for evidence) and if a single sibling is misclassified then all members of that family will incorrectly become part of the exposure discordant group. As a result, the within-family estimate may be biased. To test this, we replaced the exposure status of those not exposed to maternal smoking in pregnancy (i.e. “not exposed” was changed to “exposed”) if the mother smoked in later pregnancies. Here we assumed that it is unlikely that a mother would start smoking in later pregnancies and therefore that the earlier born individuals may be misclassified; this pattern of misclassification has been hypothesised previously (11). The family averaged exposure was recalculated using the new exposure values of each family member and the primary analyses were repeated. It should be noted that families with a sibling whose exposure was edited were no longer exposure discordant and therefore did not contribute to the within-family coefficient estimate. Further, the change in exposure status was more likely for children with lower parity than higher parity.

A5.3 - Sensitivity analysis (iii) – test of whether the pattern of smoking change across pregnancies influences the within-family estimate (ruling out carry-over effects)

It is possible that differing patterns of change in smoking status may have differing influences on the within-family estimate. Differing within-family estimates between the restricted cohorts would suggest the presence of carry-over effects (see (12) for a detailed explanation). For example the

outcome (SGA or ID) of a first born child may influence the mothers decision to smoke or use snus in subsequent pregnancies. Alternatively, the exposure to smoking during pregnancy of a second born child may influence the risk of ID in the first born child if second hand smoke influences the risk of the outcome. To test whether our results were susceptible to the influence of carry-over effects we performed a sensitivity analysis in which we restricted the sample to include only the first two members of each family included in the cohort (single children were also included in the analysis as they contribute to the between-family estimate). Analyses were run on this dataset as a whole (restricted cohort 1) and then repeated with further restriction of the exposure discordant group to only those in which the mother stopped smoking in the second pregnancy (restricted cohort 2). This was repeated again, restricting the exposure discordant group to those in which the mother started smoking in the second pregnancy (restricted cohort 3).

Restricted cohort 1 was used to check comparability with our primary analyses when using only the first two children in a family. Equivalence in effect estimates between restricted cohorts 2 and 3 would rule out carry-over effects while non-equivalence would suggest that our results may be susceptible to carry-over effects. Differing effect estimates between the restricted cohorts do not guarantee that carry-over effects are present as the difference could also be the result of unmeasured non-shared confounding or from selection bias (12).

B - Supplementary Results

B1 - Cohort descriptives

B1.1 - Descriptives separated by categories of family level smoking

		N(%)					
Variable	Level	Never smoked in pregnancy	Sometimes smoked in pregnancy	Always smoked in pregnancy	Never used snus in pregnancy	Sometimes used snus in pregnancy	Always used snus in pregnancy
Total		955928	37859	76226	1048203	13277	8533
Intellectual disability	No	950199 (99.40)	37540 (99.16)	75319 (98.81)	1041436 (99.35)	13170 (99.19)	8452 (99.05)
	Yes	5729 (0.60)	319 (0.84)	907 (1.19)	6767 (0.65)	107 (0.81)	81 (0.95)
Small for gestational age	No	934871 (97.80)	36831 (97.28)	72562 (95.19)	1022949 (97.59)	13022 (98.08)	8293 (97.19)
	Yes	18162 (1.90)	907 (2.40)	3400 (4.46)	22030 (2.10)	229 (1.72)	210 (2.46)
Sex	Female	464657 (48.61)	18422 (48.66)	36855 (48.35)	509321 (48.59)	6454 (48.61)	4159 (48.74)
	Male	491271 (51.39)	19437 (51.34)	39371 (51.65)	538882 (51.41)	6823 (51.39)	4374 (51.26)
Parity	1	431491 (45.14)	12565 (33.19)	31907 (41.86)	466955 (44.55)	4765 (35.89)	4243 (49.72)
	2	352880 (36.91)	15232 (40.23)	23435 (30.74)	383625 (36.60)	5401 (40.68)	2521 (29.54)
	3 or more	171557 (17.95)	10062 (26.58)	20884 (27.40)	197623 (18.85)	3111 (23.43)	1769 (20.73)
Highest parental education	Pre-age 16	33846 (3.54)	4099 (10.83)	11792 (15.47)	48736 (4.65)	607 (4.57)	394 (4.62)
	Age 16-18	366149 (38.30)	24275 (64.12)	52159 (68.43)	430562 (41.08)	7052 (53.11)	4969 (58.23)
	Post-age 18	555933 (58.16)	9485 (25.05)	12275 (16.10)	568905 (54.27)	5618 (42.31)	3170 (37.15)
Adjusted family income	1	98733 (10.33)	6565 (17.34)	15446 (20.26)	118509 (11.31)	1382 (10.41)	853 (10.00)
	2	186674 (19.53)	12135 (32.05)	27506 (36.08)	220468 (21.03)	3542 (26.68)	2305 (27.01)
	3	214956 (22.49)	9610 (25.38)	17691 (23.21)	236428 (22.56)	3502 (26.38)	2327 (27.27)
	4	228223 (23.87)	6431 (16.99)	10806 (14.18)	240747 (22.97)	2895 (21.80)	1818 (21.31)
	5	227342 (23.78)	3118 (8.24)	4777 (6.27)	232051 (22.14)	1956 (14.73)	1230 (14.41)

		N(%)					
Variable	Level	Never smoked in pregnancy	Sometimes smoked in pregnancy	Always smoked in pregnancy	Never used snus in pregnancy	Sometimes used snus in pregnancy	Always used snus in pregnancy
Maternal anxiety diagnosis	No	930400 (97.33)	35671 (94.22)	69961 (91.78)	1015472 (96.88)	12576 (94.72)	7984 (93.57)
	Yes	25528 (2.67)	2188 (5.78)	6265 (8.22)	32731 (3.12)	701 (5.28)	549 (6.43)
Maternal depression diagnosis	No	938174 (98.14)	36390 (96.12)	72133 (94.63)	1025798 (97.86)	12799 (96.40)	8100 (94.93)
	Yes	17754 (1.86)	1469 (3.88)	4093 (5.37)	22405 (2.14)	478 (3.60)	433 (5.07)
Maternal psychosis diagnosis	No	953064 (99.70)	37655 (99.46)	75440 (98.97)	1044510 (99.65)	13190 (99.34)	8459 (99.13)
	Yes	2864 (0.30)	204 (0.54)	786 (1.03)	3693 (0.35)	87 (0.66)	74 (0.87)
Maternal addiction diagnosis	No	945795 (98.94)	36267 (95.79)	71005 (93.15)	1032109 (98.46)	12837 (96.69)	8121 (95.17)
	Yes	10133 (1.06)	1592 (4.21)	5221 (6.85)	16094 (1.54)	440 (3.31)	412 (4.83)
Any maternal psychiatric diagnosis	No	911592 (95.36)	33818 (89.33)	64626 (84.78)	990472 (94.49)	12054 (90.79)	7510 (88.01)
	Yes	44336 (4.64)	4041 (10.67)	11600 (15.22)	57731 (5.51)	1223 (9.21)	1023 (11.99)
Any paternal psychiatric diagnosis	No	923577 (96.62)	35094 (92.70)	68242 (89.53)	1006430 (96.01)	12523 (94.32)	7960 (93.28)
	Yes	32351 (3.38)	2765 (7.30)	7984 (10.47)	41773 (3.99)	754 (5.68)	573 (6.72)
Any maternal neurodevelopmental diagnosis	No	954745 (99.88)	37697 (99.57)	75545 (99.11)	1046291 (99.82)	13227 (99.62)	8469 (99.25)
	Yes	1183 (0.12)	162 (0.43)	681 (0.89)	1912 (0.18)	50 (0.38)	64 (0.75)
Any paternal neurodevelopmental diagnosis	No	954520 (99.85)	37692 (99.56)	75544 (99.11)	1046023 (99.79)	13237 (99.70)	8496 (99.57)
	Yes	1408 (0.15)	167 (0.44)	682 (0.89)	2180 (0.21)	40 (0.30)	37 (0.43)
Maternal country of origin	Africa	28858 (3.02)	568 (1.50)	667 (0.88)	29885 (2.85)	163 (1.23)	45 (0.53)
	Americas	10344 (1.08)	310 (0.82)	516 (0.68)	11071 (1.06)	67 (0.50)	32 (0.38)
	Asia	30526 (3.19)	647 (1.71)	1161 (1.52)	32016 (3.05)	188 (1.42)	130 (1.52)
	Europe	45903 (4.80)	2755 (7.28)	6310 (8.28)	54646 (5.21)	207 (1.56)	115 (1.35)
	Middle East	53510 (5.60)	2486 (6.57)	3393 (4.45)	59033 (5.63)	278 (2.09)	78 (0.91)

Supplementary Results

		N(%)					
Variable	Level	Never smoked in pregnancy	Sometimes smoked in pregnancy	Always smoked in pregnancy	Never used snus in pregnancy	Sometimes used snus in pregnancy	Always used snus in pregnancy
	Oceania	417 (0.04)	10 (0.03)	21 (0.03)	443 (0.04)	0 (0.00)	5 (0.06)
	Scandinavia	14915 (1.56)	678 (1.79)	1905 (2.50)	17192 (1.64)	185 (1.39)	121 (1.42)
	Swedish	771455 (80.70)	30405 (80.31)	62253 (81.67)	843917 (80.51)	12189 (91.81)	8007 (93.84)
Birth year	1999-2001	202876 (21.22)	7323 (19.34)	16935 (22.22)	223270 (21.30)	2252 (16.96)	1612 (18.89)
	2002-2004	233733 (24.45)	11224 (29.65)	21867 (28.69)	260878 (24.89)	3609 (27.18)	2337 (27.39)
	2005-2007	242147 (25.33)	10401 (27.47)	18154 (23.82)	264824 (25.26)	4017 (30.26)	1861 (21.81)
	2008-2010	277172 (29.00)	8911 (23.54)	19270 (25.28)	299231 (28.55)	3399 (25.60)	2723 (31.91)
Any maternal smoking in pregnancy	No	955928 (100.00)	20107 (53.11)	0 (0.00)	956466 (91.25)	11869 (89.40)	7700 (90.24)
	Yes	0 (0.00)	17752 (46.89)	76226 (100.00)	91737 (8.75)	1408 (10.60)	833 (9.76)
Any maternal snus use in pregnancy	No	943406 (98.69)	36655 (96.82)	75287 (98.77)	1048203 (100.00)	7145 (53.81)	0 (0.00)
	Yes	12522 (1.31)	1204 (3.18)	939 (1.23)	0 (0.00)	6132 (46.19)	8533 (100.00)

B1.2 - Descriptives separated by timing of smoking exposure

		N(%)					
Variable	Level	Non-smoker before pregnancy	Smoker before pregnancy	Non-smoker at first prenatal visit	Smoker at first prenatal visit	Non-smoker at 32 weeks pregnancy	Smoker at 32 weeks pregnancy
Total		834804	189751	976351	85535	922384	58960
Intellectual disability	No	829912 (99.41)	188112 (99.14)	970439 (99.39)	84540 (98.84)	917031 (99.42)	58333 (98.94)
	Yes	4892 (0.59)	1639 (0.86)	5912 (0.61)	995 (1.16)	5353 (0.58)	627 (1.06)
Small for gestational age	No	816604 (97.82)	183344 (96.62)	954802 (97.79)	81539 (95.33)	902441 (97.84)	56213 (95.34)
	Yes	15841 (1.90)	5792 (3.05)	18623 (1.91)	3701 (4.33)	17428 (1.89)	2597 (4.40)
Sex	Female	405713 (48.60)	92092 (48.53)	474658 (48.62)	41274 (48.25)	448275 (48.60)	28638 (48.57)
	Male	429091 (51.40)	97659 (51.47)	501693 (51.38)	44261 (51.75)	474109 (51.40)	30322 (51.43)
Parity	1	356693 (42.73)	99968 (52.68)	436032 (44.66)	36231 (42.36)	412163 (44.68)	23331 (39.57)
	2	320758 (38.42)	53647 (28.27)	362401 (37.12)	26258 (30.70)	342680 (37.15)	18830 (31.94)
	3 or more	157353 (18.85)	36136 (19.04)	177918 (18.22)	23046 (26.94)	167541 (18.16)	16799 (28.49)
Highest parental education	Pre-age 16	28039 (3.36)	19545 (10.30)	36553 (3.74)	12777 (14.94)	34074 (3.69)	9307 (15.79)
	Age 16-18	301612 (36.13)	119674 (63.07)	380706 (38.99)	58409 (68.29)	355446 (38.54)	40328 (68.40)
	Post-age 18	505153 (60.51)	50532 (26.63)	559092 (57.26)	14349 (16.78)	532864 (57.77)	9325 (15.82)
Adjusted family income	1	84870 (10.17)	30060 (15.84)	102812 (10.53)	16849 (19.70)	95327 (10.33)	12081 (20.49)
	2	162156 (19.42)	54673 (28.81)	194396 (19.91)	30254 (35.37)	183600 (19.90)	21725 (36.85)
	3	186645 (22.36)	45377 (23.91)	220568 (22.59)	19971 (23.35)	208840 (22.64)	13670 (23.19)
	4	198245 (23.75)	36880 (19.44)	231046 (23.66)	12670 (14.81)	218766 (23.72)	8107 (13.75)
	5	202888 (24.30)	22761 (12.00)	227529 (23.30)	5791 (6.77)	215851 (23.40)	3377 (5.73)
Maternal anxiety diagnosis	No	813114 (97.40)	177949 (93.78)	949283 (97.23)	78830 (92.16)	895717 (97.11)	53959 (91.52)
	Yes	21690 (2.60)	11802 (6.22)	27068 (2.77)	6705 (7.84)	26667 (2.89)	5001 (8.48)

Supplementary Results

		N(%)					
Variable	Level	Non-smoker before pregnancy	Smoker before pregnancy	Non-smoker at first prenatal visit	Smoker at first prenatal visit	Non-smoker at 32 weeks pregnancy	Smoker at 32 weeks pregnancy
Maternal depression diagnosis	No	819815 (98.20)	181724 (95.77)	957612 (98.08)	81103 (94.82)	903863 (97.99)	55679 (94.44)
	Yes	14989 (1.80)	8027 (4.23)	18739 (1.92)	4432 (5.18)	18521 (2.01)	3281 (5.56)
Maternal psychosis diagnosis	No	832385 (99.71)	188416 (99.30)	973347 (99.69)	84711 (99.04)	919459 (99.68)	58389 (99.03)
	Yes	2419 (0.29)	1335 (0.70)	3004 (0.31)	824 (0.96)	2925 (0.32)	571 (0.97)
Maternal addiction diagnosis	No	827232 (99.09)	180681 (95.22)	965108 (98.85)	79965 (93.49)	911266 (98.79)	55006 (93.29)
	Yes	7572 (0.91)	9070 (4.78)	11243 (1.15)	5570 (6.51)	11118 (1.21)	3954 (6.71)
Any maternal psychiatric diagnosis	No	797753 (95.56)	167805 (88.43)	929257 (95.18)	73039 (85.39)	876077 (94.98)	49894 (84.62)
	Yes	37051 (4.44)	21946 (11.57)	47094 (4.82)	12496 (14.61)	46307 (5.02)	9066 (15.38)
Any paternal psychiatric diagnosis	No	807443 (96.72)	174872 (92.16)	942125 (96.49)	76944 (89.96)	888907 (96.37)	52704 (89.39)
	Yes	27361 (3.28)	14879 (7.84)	34226 (3.51)	8591 (10.04)	33477 (3.63)	6256 (10.61)
Any maternal neurodevelopmental diagnosis	No	833882 (99.89)	188669 (99.43)	975041 (99.87)	84837 (99.18)	921055 (99.86)	58432 (99.10)
	Yes	922 (0.11)	1082 (0.57)	1310 (0.13)	698 (0.82)	1329 (0.14)	528 (0.90)
Any paternal neurodevelopmental diagnosis	No	833706 (99.87)	188609 (99.40)	974802 (99.84)	84838 (99.19)	920835 (99.83)	58424 (99.09)
	Yes	1098 (0.13)	1142 (0.60)	1549 (0.16)	697 (0.81)	1549 (0.17)	536 (0.91)
Maternal country of origin	Africa	26877 (3.22)	1967 (1.04)	29108 (2.98)	774 (0.90)	27623 (2.99)	503 (0.85)
	Americas	8308 (1.00)	2409 (1.27)	10498 (1.08)	583 (0.68)	9699 (1.05)	359 (0.61)
	Asia	27737 (3.32)	3564 (1.88)	30853 (3.16)	1280 (1.50)	29511 (3.20)	855 (1.45)
	Europe	38593 (4.62)	14498 (7.64)	47810 (4.90)	6788 (7.94)	45202 (4.90)	4938 (8.38)
	Middle East	48724 (5.84)	8328 (4.39)	55173 (5.65)	3778 (4.42)	51903 (5.63)	2773 (4.70)
	Oceania	368 (0.04)	60 (0.03)	419 (0.04)	24 (0.03)	391 (0.04)	15 (0.03)
	Scandinavia	12864 (1.54)	3739 (1.97)	15279 (1.56)	2066 (2.42)	14229 (1.54)	1347 (2.28)
	Swedish	671333 (80.42)	155186 (81.78)	787211 (80.63)	70242 (82.12)	743826 (80.64)	48170 (81.70)

Supplementary Results

		N(%)					
Variable	Level	Non-smoker before pregnancy	Smoker before pregnancy	Non-smoker at first prenatal visit	Smoker at first prenatal visit	Non-smoker at 32 weeks pregnancy	Smoker at 32 weeks pregnancy
Birth year	1999-2001	147872 (17.71)	39089 (20.60)	204846 (20.98)	19224 (22.48)	152383 (16.52)	10976 (18.62)
	2002-2004	210815 (25.25)	53438 (28.16)	239054 (24.48)	25317 (29.60)	236712 (25.66)	17628 (29.90)
	2005-2007	220926 (26.46)	48280 (25.44)	248595 (25.46)	20604 (24.09)	247917 (26.88)	15097 (25.61)
	2008-2010	255191 (30.57)	48944 (25.79)	283856 (29.07)	20390 (23.84)	285372 (30.94)	15259 (25.88)
Any maternal smoking in pregnancy	No	831553 (99.61)	100033 (52.72)	968612 (99.21)	0 (0.00)	903165 (97.92)	0 (0.00)
	Yes	3251 (0.39)	89718 (47.28)	7739 (0.79)	85535 (100.00)	19219 (2.08)	58960 (100.00)
Any maternal snus use in pregnancy	No	822889 (98.57)	187165 (98.64)	962966 (98.63)	84484 (98.77)	909740 (98.63)	58147 (98.62)
	Yes	11915 (1.43)	2586 (1.36)	13385 (1.37)	1051 (1.23)	12644 (1.37)	813 (1.38)

B1.3 - Descriptives separated by timing of snus exposure

		N(%)					
Variable	Level	Non-snus before pregnancy	Snus before pregnancy	Non-snus at first prenatal visit	Snus at first prenatal visit	Non-snus at 32 weeks	Snus at 32 weeks
Total		993624	27731	1051126	12837	1061814	4945
Intellectual disability	No	987317 (99.37)	27557 (99.37)	1044325 (99.35)	12732 (99.18)	1054943 (99.35)	4886 (98.81)
	Yes	6307 (0.63)	174 (0.63)	6801 (0.65)	105 (0.82)	6871 (0.65)	59 (1.19)
Small for gestational age	No	969713 (97.59)	27112 (97.77)	1025859 (97.60)	12517 (97.51)	1036291 (97.60)	4830 (97.67)
	Yes	21035 (2.12)	536 (1.93)	22074 (2.10)	282 (2.20)	22274 (2.10)	99 (2.00)
Sex	Female	482675 (48.58)	13520 (48.75)	510751 (48.59)	6216 (48.42)	516017 (48.60)	2419 (48.92)
	Male	510949 (51.42)	14211 (51.25)	540375 (51.41)	6621 (51.58)	545797 (51.40)	2526 (51.08)
Parity	1	439804 (44.26)	14873 (53.63)	467614 (44.49)	5496 (42.81)	472638 (44.51)	1944 (39.31)
	2	365269 (36.76)	8369 (30.18)	385038 (36.63)	4415 (34.39)	388596 (36.60)	1831 (37.03)
	3 or more	188551 (18.98)	4489 (16.19)	198474 (18.88)	2926 (22.79)	200580 (18.89)	1170 (23.66)
Highest parental education	Pre-age 16	46475 (4.68)	812 (2.93)	48830 (4.65)	554 (4.32)	49343 (4.65)	245 (4.95)
	Age 16-18	405277 (40.79)	13792 (49.73)	432629 (41.16)	7309 (56.94)	437858 (41.24)	2921 (59.07)
	Post-age 18	541872 (54.53)	13127 (47.34)	569667 (54.20)	4974 (38.75)	574613 (54.12)	1779 (35.98)
Adjusted family income	1	112327 (11.30)	2023 (7.30)	118677 (11.29)	1222 (9.52)	119886 (11.29)	533 (10.78)
	2	209786 (21.11)	6392 (23.05)	221608 (21.08)	3494 (27.22)	224040 (21.10)	1404 (28.39)
	3	223969 (22.54)	7362 (26.55)	237458 (22.59)	3517 (27.40)	240002 (22.60)	1386 (28.03)
	4	227470 (22.89)	6929 (24.99)	241407 (22.97)	2785 (21.70)	243788 (22.96)	989 (20.00)
	5	220072 (22.15)	5025 (18.12)	231976 (22.07)	1819 (14.17)	234098 (22.05)	633 (12.80)
Maternal anxiety diagnosis	No	961733 (96.79)	26186 (94.43)	1018129 (96.86)	12035 (93.75)	1028373 (96.85)	4610 (93.23)
	Yes	31891 (3.21)	1545 (5.57)	32997 (3.14)	802 (6.25)	33441 (3.15)	335 (6.77)

Supplementary Results

		N(%)					
Variable	Level	Non-snus before pregnancy	Snus before pregnancy	Non-snus at first prenatal visit	Snus at first prenatal visit	Non-snus at 32 weeks	Snus at 32 weeks
Maternal depression diagnosis	No	971839 (97.81)	26529 (95.67)	1028524 (97.85)	12241 (95.36)	1038902 (97.84)	4687 (94.78)
	Yes	21785 (2.19)	1202 (4.33)	22602 (2.15)	596 (4.64)	22912 (2.16)	258 (5.22)
Maternal psychosis diagnosis	No	990078 (99.64)	27539 (99.31)	1047393 (99.64)	12740 (99.24)	1058040 (99.64)	4888 (98.85)
	Yes	3546 (0.36)	192 (0.69)	3733 (0.36)	97 (0.76)	3774 (0.36)	57 (1.15)
Maternal addiction diagnosis	No	978014 (98.43)	26750 (96.46)	1034849 (98.45)	12292 (95.75)	1045262 (98.44)	4697 (94.98)
	Yes	15610 (1.57)	981 (3.54)	16277 (1.55)	545 (4.25)	16552 (1.56)	248 (5.02)
Any maternal psychiatric diagnosis	No	937575 (94.36)	24906 (89.81)	992921 (94.46)	11400 (88.81)	1002824 (94.44)	4329 (87.54)
	Yes	56049 (5.64)	2825 (10.19)	58205 (5.54)	1437 (11.19)	58990 (5.56)	616 (12.46)
Any paternal psychiatric diagnosis	No	953049 (95.92)	26168 (94.36)	1009087 (96.00)	12036 (93.76)	1019284 (95.99)	4590 (92.82)
	Yes	40575 (4.08)	1563 (5.64)	42039 (4.00)	801 (6.24)	42530 (4.01)	355 (7.18)
Any maternal neurodevelopmental diagnosis	No	991739 (99.81)	27617 (99.59)	1049196 (99.82)	12760 (99.40)	1059846 (99.81)	4906 (99.21)
	Yes	1885 (0.19)	114 (0.41)	1930 (0.18)	77 (0.60)	1968 (0.19)	39 (0.79)
Any paternal neurodevelopmental diagnosis	No	991481 (99.78)	27637 (99.66)	1048919 (99.79)	12793 (99.66)	1059589 (99.79)	4925 (99.60)
	Yes	2143 (0.22)	94 (0.34)	2207 (0.21)	44 (0.34)	2225 (0.21)	20 (0.40)
Maternal country of origin	Africa	28715 (2.89)	137 (0.49)	29848 (2.84)	91 (0.71)	30045 (2.83)	29 (0.59)
	Americas	10553 (1.06)	95 (0.34)	11044 (1.05)	50 (0.39)	11138 (1.05)	19 (0.38)
	Asia	30941 (3.11)	341 (1.23)	32020 (3.05)	178 (1.39)	32227 (3.04)	64 (1.29)
	Europe	52601 (5.29)	296 (1.07)	54517 (5.19)	152 (1.18)	54855 (5.17)	71 (1.44)
	Middle East	56717 (5.71)	223 (0.80)	58919 (5.61)	143 (1.11)	59296 (5.58)	51 (1.03)
	Oceania	415 (0.04)	10 (0.04)	439 (0.04)	5 (0.04)	446 (0.04)	1 (0.02)
	Scandinavia	16165 (1.63)	364 (1.31)	17206 (1.64)	177 (1.38)	17373 (1.64)	78 (1.58)
	Swedish	797517 (80.26)	26265 (94.71)	847133 (80.59)	12041 (93.80)	856434 (80.66)	4632 (93.67)

Supplementary Results

		N(%)					
Variable	Level	Non-snus before pregnancy	Snus before pregnancy	Non-snus at first prenatal visit	Snus at first prenatal visit	Non-snus at 32 weeks	Snus at 32 weeks
Birth year	1999-2001	180039 (18.12)	3763 (13.57)	223232 (21.24)	2076 (16.17)	225591 (21.25)	777 (15.71)
	2002-2004	257362 (25.90)	6900 (24.88)	261301 (24.86)	3623 (28.22)	264542 (24.91)	1419 (28.70)
	2005-2007	262130 (26.38)	7082 (25.54)	265937 (25.30)	3306 (25.75)	268921 (25.33)	1135 (22.95)
	2008-2010	294093 (29.60)	9986 (36.01)	300656 (28.60)	3832 (29.85)	302760 (28.51)	1614 (32.64)
Any maternal smoking	No	902819 (90.86)	26321 (94.92)	959456 (91.28)	11739 (91.45)	968846 (91.24)	4458 (90.15)
in pregnancy	Yes	90805 (9.14)	1410 (5.08)	91670 (8.72)	1098 (8.55)	92968 (8.76)	487 (9.85)
Any maternal snus use	No	990592 (99.69)	16188 (58.38)	1049436 (99.84)	0 (0.00)	1055348 (99.39)	0 (0.00)
in pregnancy	Yes	3032 (0.31)	11543 (41.62)	1690 (0.16)	12837 (100.00)	6466 (0.61)	4945 (100.00)

B1.4 - Descriptives of cohort for sensitivity analysis (ii) separated by change in smoking behaviour across first two pregnancies

		N(%)		
Variable	Level	Total	Stopped	Started
Total		660000	8877	4960
Intellectual disability	No	655311 (99.29)	8809 (99.23)	4913 (99.05)
	Yes	4689 (0.71)	68 (0.77)	47 (0.95)
Small for gestational age	No	640357 (97.02)	8492 (95.66)	4806 (96.90)
	Yes	17368 (2.63)	347 (3.91)	132 (2.66)
Sex	Female	320440 (48.55)	4274 (48.15)	2428 (48.95)
	Male	339560 (51.45)	4603 (51.85)	2532 (51.05)
Parity	1	459529 (69.63)	7417 (83.55)	3795 (76.51)
	2	116616 (17.67)	933 (10.51)	741 (14.94)
	3 or more	83855 (12.71)	527 (5.94)	424 (8.55)
Highest parental education	Pre-age 16	32179 (4.88)	852 (9.60)	727 (14.66)
	Age 16-18	278608 (42.21)	5792 (65.25)	3094 (62.38)
	Post-age 18	349213 (52.91)	2233 (25.15)	1139 (22.96)
Adjusted family income	1	78120 (11.84)	1402 (15.79)	1221 (24.62)
	2	124325 (18.84)	2057 (23.17)	1210 (24.40)
	3	129135 (19.57)	2008 (22.62)	928 (18.71)
	4	158898 (24.08)	2149 (24.21)	1026 (20.69)
	5	169522 (25.69)	1261 (14.21)	575 (11.59)
Maternal anxiety diagnosis	No	640695 (97.07)	8537 (96.17)	4806 (96.90)
	Yes	19305 (2.92)	340 (3.83)	154 (3.10)
Maternal depression diagnosis	No	646562 (97.96)	8651 (97.45)	4854 (97.86)
	Yes	13438 (2.04)	226 (2.55)	106 (2.14)
Maternal psychosis diagnosis	No	657605 (99.64)	8847 (99.66)	4941 (99.62)
	Yes	2395 (0.36)	30 (0.34)	19 (0.38)
Maternal addiction diagnosis	No	649328 (98.38)	8514 (95.91)	4812 (97.02)
	Yes	10672 (1.62)	363 (4.09)	148 (2.98)
Any maternal psychiatric diagnosis	No	625008 (94.70)	8144 (91.74)	4622 (93.19)
	Yes	34992 (5.30)	733 (8.26)	338 (6.81)
Any paternal psychiatric diagnosis	No	634255 (96.10)	8316 (93.68)	4691 (94.58)
	Yes	25745 (3.90)	561 (6.32)	269 (5.42)
Any maternal neurodevelopmental diagnosis	No	658681 (99.80)	8860 (99.81)	4940 (99.60)
	Yes	1319 (0.20)	17 (0.19)	20 (0.40)
	No	658564 (99.78)	8849 (99.68)	4943 (99.66)

Supplementary Results

		N(%)		
Variable	Level	Total	Stopped	Started
Any paternal neurodevelopmental diagnosis	Yes	1436 (0.22)	28 (0.32)	17 (0.34)
Maternal country of origin	Africa	17344 (2.63)	83 (0.94)	85 (1.71)
	Americas	7414 (1.12)	50 (0.56)	50 (1.01)
	Asia	22203 (3.36)	137 (1.54)	105 (2.12)
	Europe	37087 (5.62)	585 (6.59)	443 (8.93)
	Middle East	36437 (5.52)	340 (3.83)	456 (9.19)
	Oceania	282 (0.04)	0 (0.00)	2 (0.04)
	Scandinavia	11139 (1.69)	157 (1.77)	87 (1.75)
	Swedish	528094 (80.01)	7525 (84.77)	3732 (75.24)
Birth year	1999-2001	203251 (30.80)	3004 (33.84)	2320 (46.77)
	2002-2004	162743 (24.66)	3686 (41.52)	1418 (28.59)
	2005-2007	139735 (21.17)	1803 (20.31)	1014 (20.44)
	2008-2010	154271 (23.37)	384 (4.33)	208 (4.19)

B2 - Missing data

B2.1 - Descriptives for missing covariate data

Variable	Level	Included, N(%)	Excluded, N(%)	O.R. for exclusion (95% CI)
Total		1132473 (100.00)	824 (100.00)	
Intellectual disability	No	1124940 (99.33)	811 (98.42)	Ref
	Yes	7533 (0.67)	13 (1.58)	2.39 (1.38-4.14)
Small for gestational	No	1104238 (97.51)	785 (95.27)	Ref
age	Yes	24090 (2.13)	37 (4.49)	2.16 (1.55-3.01)
Any maternal	No	976053 (86.19)	696 (84.47)	Ref
smoking in pregnancy	Yes	103099 (9.10)	80 (9.71)	1.09 (0.86-1.37)
	Missing	53321 (4.71)	48 (5.83)	1.26 (0.94-1.69)
Any maternal snus	No	1055533 (93.21)	762 (92.48)	Ref
use in pregnancy	Yes	14972 (1.32)	9 (1.09)	0.83 (0.43-1.61)
	Missing	61968 (5.47)	53 (6.43)	1.18 (0.90-1.57)
Sex	Female	549959 (48.56)	402 (48.79)	Ref
	Male	582514 (51.44)	422 (51.21)	0.99 (0.86-1.14)
Parity	1	503786 (44.49)	393 (47.69)	Ref
	2	413596 (36.52)	183 (22.21)	0.57 (0.48-0.68)
	3 or more	215091 (18.99)	248 (30.10)	1.48 (1.26-1.73)
Highest parental	Pre-age 16	53744 (4.75)	19 (2.31)	Ref
education	Age 16-18	467625 (41.29)	40 (4.85)	0.24 (0.14-0.42)
	Post-age 18	611104 (53.96)	72 (8.74)	0.33 (0.20-0.55)
Adjusted family	1	128677 (11.36)	430 (52.18)	19.75 (14.44-27.03)
income	2	238887 (21.09)	161 (19.54)	3.98 (2.85-5.58)
	3	254172 (22.44)	43 (5.22)	Ref
	4	257905 (22.77)	20 (2.43)	0.46 (0.27-0.78)
	5	252832 (22.33)	61 (7.40)	1.43 (0.97-2.11)
Maternal anxiety	No	1096769 (96.85)	804 (97.57)	Ref
diagnosis	Yes	35704 (3.15)	20 (2.43)	0.76 (0.49-1.19)
Maternal depression	No	1108057 (97.84)	804 (97.57)	Ref
diagnosis	Yes	24416 (2.16)	20 (2.43)	1.13 (0.72-1.76)
Maternal psychosis	No	1128347 (99.64)	822 (99.76)	Ref
diagnosis	Yes	4126 (0.36)	2 (0.24)	0.67 (0.17-2.67)
Maternal addiction	No	1114428 (98.41)	821 (99.64)	Ref
diagnosis	Yes	18045 (1.59)	3 (0.36)	0.23 (0.07-0.70)
Any maternal	No	1069221 (94.41)	788 (95.63)	Ref
psychiatric diagnosis	Yes	63252 (5.59)	36 (4.37)	0.77 (0.55-1.08)
Any paternal	No	1087032 (95.99)	806 (97.82)	Ref
psychiatric diagnosis	Yes	45441 (4.01)	18 (2.18)	0.53 (0.33-0.85)
	No	1130342 (99.81)	816 (99.03)	Ref

Supplementary Results

Variable	Level	Included, N(%)	Excluded, N(%)	O.R. for exclusion (95% CI)
Any maternal neurodevelopmental diagnosis	Yes	2131 (0.19)	8 (0.97)	5.20 (2.59-10.45)
Any paternal neurodevelopmental diagnosis	No	1130111 (99.79)	822 (99.76)	Ref
	Yes	2362 (0.21)	2 (0.24)	1.16 (0.29-4.67)
Maternal country of origin	Africa	31815 (2.81)	153 (18.57)	84.64 (61.78-115.96)
	Americas	11914 (1.05)	16 (1.94)	23.64 (13.49-41.41)
	Asia	33954 (3.00)	71 (8.62)	36.80 (25.73-52.64)
	Europe	57821 (5.11)	137 (16.63)	41.70 (30.30-57.39)
	Middle East	62334 (5.50)	93 (11.29)	26.26 (18.70-36.87)
	Oceania	491 (0.04)	4 (0.49)	143.38 (51.66-397.96)
	Scandinavia	18925 (1.67)	59 (7.16)	54.87 (37.78-79.68)
	Swedish	915219 (80.82)	52 (6.31)	Ref
Birth year	1999-2001	250416 (22.11)	85 (10.32)	Ref
	2002-2004	276150 (24.38)	128 (15.53)	1.37 (1.04-1.80)
	2005-2007	293076 (25.88)	214 (25.97)	2.15 (1.67-2.77)
	2008-2010	312831 (27.62)	397 (48.18)	3.74 (2.96-4.73)

B2.2 - Descriptives for missing exposure data

Variable	Level	Included, N(%)	Excluded, N(%)	O.R. for exclusion (95% CI)
Total		1070013 (100.00)	62460 (100.00)	
Intellectual disability	No	1063058 (99.35)	61882 (99.07)	Ref
	Yes	6955 (0.65)	578 (0.93)	1.43 (1.31-1.55)
Small for gestational age	No	1044264 (97.59)	59974 (96.02)	Ref
	Yes	22469 (2.10)	1621 (2.60)	1.26 (1.19-1.32)
Any maternal smoking in pregnancy	No	976035 (91.22)	18 (0.03)	Ref
	Yes	93978 (8.78)	9121 (14.60)	5262.71 (3314.06-8357.15)
	Missing	0 (0.00)	53321 (85.37)	
Any maternal snus use in pregnancy	No	1055348 (98.63)	185 (0.30)	Ref
	Yes	14665 (1.37)	307 (0.49)	119.42 (99.44-143.42)
	Missing	0 (0.00)	61968 (99.21)	
Sex	Female	519934 (48.59)	30025 (48.07)	Ref
	Male	550079 (51.41)	32435 (51.93)	1.02 (1.00-1.04)
Parity	1	475963 (44.48)	27823 (44.55)	Ref
	2	391547 (36.59)	22049 (35.30)	0.96 (0.95-0.98)
	3 or more	202503 (18.93)	12588 (20.15)	1.06 (1.04-1.09)
Highest parental education	Pre-age 16	49737 (4.65)	4007 (6.42)	Ref
	Age 16-18	442583 (41.36)	25042 (40.09)	0.70 (0.68-0.73)
	Post-age 18	577693 (53.99)	33411 (53.49)	0.72 (0.69-0.74)
Adjusted family income	1	120744 (11.28)	7933 (12.70)	1.34 (1.30-1.38)
	2	226315 (21.15)	12572 (20.13)	1.13 (1.10-1.16)
	3	242257 (22.64)	11915 (19.08)	Ref
	4	245460 (22.94)	12445 (19.92)	1.03 (1.00-1.06)
	5	235237 (21.98)	17595 (28.17)	1.52 (1.48-1.56)
Maternal anxiety diagnosis	No	1036032 (96.82)	60737 (97.24)	Ref
	Yes	33981 (3.18)	1723 (2.76)	0.86 (0.82-0.91)
Maternal depression diagnosis	No	1046697 (97.82)	61360 (98.24)	Ref
	Yes	23316 (2.18)	1100 (1.76)	0.80 (0.76-0.86)
Maternal psychosis diagnosis	No	1066159 (99.64)	62188 (99.56)	Ref
	Yes	3854 (0.36)	272 (0.44)	1.21 (1.07-1.37)
Maternal addiction diagnosis	No	1053067 (98.42)	61361 (98.24)	Ref
	Yes	16946 (1.58)	1099 (1.76)	1.11 (1.05-1.18)
Any maternal psychiatric diagnosis	No	1010036 (94.39)	59185 (94.76)	Ref
	Yes	59977 (5.61)	3275 (5.24)	0.93 (0.90-0.97)
Any paternal psychiatric diagnosis	No	1026913 (95.97)	60119 (96.25)	Ref
	Yes	43100 (4.03)	2341 (3.75)	0.93 (0.89-0.97)
	No	1067987 (99.81)	62355 (99.83)	Ref

Supplementary Results

Variable	Level	Included, N(%)	Excluded, N(%)	O.R. for exclusion (95% CI)
Any maternal neurodevelopmental diagnosis	Yes	2026 (0.19)	105 (0.17)	0.89 (0.73-1.08)
Any paternal	No	1067756 (99.79)	62355 (99.83)	Ref
neurodevelopmental diagnosis	Yes	2257 (0.21)	105 (0.17)	0.80 (0.65-0.97)
Maternal country of origin	Africa	30093 (2.81)	1722 (2.76)	0.97 (0.92-1.02)
	Americas	11170 (1.04)	744 (1.19)	1.13 (1.05-1.21)
	Asia	32334 (3.02)	1620 (2.59)	0.85 (0.81-0.89)
	Europe	54968 (5.14)	2853 (4.57)	0.88 (0.84-0.91)
	Middle East	59389 (5.55)	2945 (4.72)	0.84 (0.81-0.87)
	Oceania	448 (0.04)	43 (0.07)	1.62 (1.19-2.22)
	Scandinavia	17498 (1.64)	1427 (2.28)	1.38 (1.31-1.46)
	Swedish	864113 (80.76)	51106 (81.82)	Ref
Birth year	1999-2001	227134 (21.23)	23282 (37.28)	Ref
	2002-2004	266824 (24.94)	9326 (14.93)	0.34 (0.33-0.35)
	2005-2007	270702 (25.30)	22374 (35.82)	0.81 (0.79-0.82)
	2008-2010	305353 (28.54)	7478 (11.97)	0.24 (0.23-0.25)

B3 - Sensitivity analyses

B3.1 - Sensitivity analysis (i) – influence of using a cleaner comparison group

Repetition of the primary analyses, excluding those in the comparison group with exposure to nicotine, are presented in Table B3.1 for ID and Table B3.2 for SGA. These analyses show very little difference to the primary analyses for the smoking-ID, smoking-SGA and snus-ID associations. Exposure to smoking and snus during pregnancy are associated with increased odds of ID in conventional models but within-between models show that this association is driven by residual confounding. Exposure to smoking during pregnancy still shows an association of increased odds of having a child born SGA that appears to be driven by a mix of within-family and between family effects. This suggests that the conventional models are still subject to residual confounding but that smoking during pregnancy does have a causal effect on the risk of offspring being born SGA. Using the cleaner comparison group, an association of increased odds of offspring born SGA was found for exposure to snus use during pregnancy in conventional unadjusted and adjusted models. This association was not observed in primary analyses. Within-between models using the cleaner comparison group were not able to determine if these associations were the result of causal effects of residual confounding.

Repetition of the secondary analyses of exposure timing, excluding those in the comparison group with exposure to nicotine at any time, are presented in Table B3.3 for ID and Table B3.4 for SGA. The results of the sensitivity analyses of exposure timing did not materially differ from the main analyses for any exposure-outcome combination.

B3.2 - Sensitivity analysis (ii) – influence of measurement error in the exposure variable among exposure discordant families

In our second sensitivity analysis we tested whether our results would be influenced by exposure misclassification in the exposure discordant group. The results are presented in Table B3.5. There were 7078 individuals who were reported as not exposed to smoking and had their exposure status changed (0.73% of unexposed individuals). This led to a total of 13 093 individuals (34.58% of exposure discordant individuals) from 5507 families (35.39% of exposure discordant families) who had been classed as exposure discordant who subsequently became exposure concordant in the sensitivity analysis. Comparison of the results of the sensitivity analysis with the primary analysis shows that occurrence of the hypothetical extreme situation, in which all unexposed individuals with exposed younger siblings were misclassified, would only influence the results of the SGA within-between model that was not adjusted for confounders (the within-family estimate increased in

magnitude while the between-family estimate became closer to 1). No other models showed notable changes to parameter estimates.

B3.3 - Sensitivity analysis (iii) – test of whether the pattern of smoking change across pregnancies influences the within-family estimate (ruling out carry-over effects)

In the third sensitivity analysis we tested whether there were differing effects for those who started smoking between pregnancies versus those who stopped smoking between pregnancies by applying different restrictions to the cohort. Briefly recapping these restrictions, restricted cohort 1 limited the cohort sample to the first two siblings in a family. Restricted cohort 2 limited the exposure discordant group in restricted cohort 1 to only those who stopped smoking between the two pregnancies. Restricted cohort 3 restricted the exposure discordant group in restricted cohort 1 to only those who started smoking between the two pregnancies.

The exposure discordant group in restricted cohort 2 contained 17 695 children with 131 cases of ID and 467 cases of SGA. The exposure discordant group in restricted cohort 3 contained 9920 children with 84 cases of ID and 238 cases of SGA.

Table B3.6 shows that the results of restricted cohort 1 are similar to the primary analyses for both ID and SGA, showing that restricting the cohort to the first two available siblings did not substantially change results. The table also shows that the different patterns of change in smoking across pregnancies gave rise to substantially overlapping confidence intervals for the within-family estimates for the outcome ID. We therefore do not consider there to be any difference and can rule out carry-over effects for the outcome ID.

Comparing the effect estimates of Model 3 for offspring SGA across restricted cohorts 2 and 3, those whose mother stopped smoking between pregnancies (restricted cohort 2) had a within-family effect estimate showing increased odds of SGA and a null between-family effect estimate. In contrast, those whose mother started smoking between pregnancies (restricted cohort 3) had a null within-family effect estimate and a between-family effect estimate showing increased odds of offspring SGA. The interpretation of the estimates across the two restricted cohorts would be that we have provided evidence for a causal effect (among the exposure discordant subgroup) of smoking in pregnancy on SGA for those who stop smoking during pregnancy but not those who start smoking during pregnancy. We are therefore unable to rule out carry-over effects for the outcome SGA though we note that the difference between the two cohorts disappeared once the within-between model was adjusted for measured confounders.

Supplementary Results

Table B3.1: Sensitivity analysis of the association between exposure and offspring ID using a cleaner comparison group.

		Smoking in pregnancy		Snus use in pregnancy	
Model	Coefficient	O.R. [a]	95% CI	O.R. [b]	95% CI
1 - Conventional unadjusted [c]	Population-averaged	1.81	(1.69-1.94)	1.46	(1.22-1.75)
2 - Conventional adjusted [d]	Population-averaged	1.25	(1.16-1.34)	1.31	(1.10-1.57)
3 - Within-between unadjusted [c, e]	Within-family	0.88	(0.71-1.10)	0.95	(0.62-1.44)
	Between-family	2.22	(1.76-2.80)	1.71	(1.08-2.70)
4 - Within-between adjusted [d, e]	Within-family	0.89	(0.72-1.11)	0.92	(0.60-1.42)
	Between-family	1.46	(1.16-1.84)	1.53	(0.96-2.45)

[a] Estimates produced using a total sample size of 1 056 712 individuals from 697 421 families including 6847 cases of ID.

[b] Estimates produced using a total sample size of 977 399 individuals from 646 915 families including 5919 cases of ID.

[c] Model adjusted for year of birth.

[d] Model adjusted for year of birth, sex, parity, highest parental education, income, parental psychiatric history, maternal country of origin and maternal age at birth.

[e] Model adjusted for family averaged exposure.

Table B3.2: Sensitivity analysis of the association between exposure and offspring SGA using a cleaner comparison group.

		Smoking in pregnancy		Snus use in pregnancy	
Model	Coefficient	O.R. [a]	95% CI	O.R. [b]	95% CI
1 - Conventional unadjusted	Population-averaged	2.26	(2.18-2.35)	1.14	(1.02-1.28)
2 - Conventional adjusted [c]	Population-averaged	2.19	(2.11-2.28)	1.22	(1.09-1.37)
3 - Within-between unadjusted [d]	Within-family	1.66	(1.48-1.87)	1.04	(0.81-1.33)
	Between-family	1.41	(1.25-1.60)	1.13	(0.85-1.49)
4 - Within-between adjusted [c, d]	Within-family	1.43	(1.26-1.62)	1.06	(0.81-1.38)
	Between-family	1.62	(1.42-1.85)	1.19	(0.89-1.60)

[a] Estimates produced using a total sample size of 1 053 467 individuals from 696 069 families including 22 211 cases of SGA.

[b] Estimates produced using a total sample size of 974 434 individuals from 645 666 families including 18 563 cases of SGA.

[c] Model adjusted for year of birth, sex, parity, highest parental education, income, parental psychiatric history, maternal country of origin and maternal age at birth.

[d] Model adjusted for family averaged exposure.

Supplementary Results

Table B3.3: Sensitivity analysis of the association between exposure timing and offspring ID using a cleaner comparison group.

		Smoking in pregnancy		Snus use in pregnancy	
Model	Coefficient	O.R. [a]	95% CI	O.R. [b]	95% CI
1 - Conventional unadjusted [c]	Non-user	1.00		1.00	
(population-averaged estimates)	User before pregnancy only	1.03	(0.95-1.12)	0.77	(0.59-0.99)
	Quit during pregnancy	1.39	(1.18-1.64)	1.15	(0.81-1.65)
	Used late into pregnancy	1.78	(1.63-1.94)	2.19	(1.59-3.04)
2 - Conventional adjusted [c, d]	Non-user	1.00		1.00	
(population-averaged estimates)	User before pregnancy only	0.89	(0.82-0.97)	0.82	(0.63-1.07)
	Quit during pregnancy	1.04	(0.88-1.22)	1.09	(0.76-1.57)
	Used late into pregnancy	1.16	(1.06-1.27)	1.88	(1.36-2.61)
3 - Unadjusted conditional logistic [c]	Non-user	1.00		1.00	
(within-family estimates)	User before pregnancy only	0.89	(0.72-1.11)	0.79	(0.46-1.36)
	Quit during pregnancy	0.87	(0.59-1.30)	0.42	(0.17-1.01)
	Used late into pregnancy	0.93	(0.65-1.34)	0.96	(0.37-2.53)
4 - Adjusted conditional logistic [c, d]	Non-user	1.00		1.00	
(within-family estimates)	User before pregnancy only	0.86	(0.69-1.08)	0.86	(0.49-1.50)
	Quit during pregnancy	0.85	(0.57-1.28)	0.39	(0.16-0.96)
	Used late into pregnancy	0.87	(0.60-1.26)	0.90	(0.34-2.39)

[a] Estimates conventional models produced using a total sample size of 1 020 188 individuals from 678 197 families including 6466 cases of ID. Estimates for conditional logistic models produced using a total sample size of 8064 individuals including 3469 cases of ID.

[b] Estimates for conventional models produced using a total sample size of 871 565 individuals from 584 447 families including 6466 cases of ID. Estimates for conditional logistic models produced using a total sample size of 6426 individuals including 3469 cases of ID.

[c] Model adjusted for year of birth.

[d] Model adjusted for year of birth, sex, parity, highest parental education, income, parental psychiatric history, maternal country of origin and maternal age at birth.

Table B3.4: Sensitivity analysis of the association between exposure timing and offspring SGA using a cleaner comparison group.

		Smoking in pregnancy		Snus use in pregnancy	
Model	Coefficient	O.R. [a]	95% CI	O.R. [b]	95% CI
1 - Conventional unadjusted (population-averaged estimates)	Non-user	1.00		1.00	
	User before pregnancy only	1.04	(0.99-1.09)	0.89	(0.78-1.01)
	Quit during pregnancy	1.44	(1.32-1.58)	1.00	(0.81-1.24)
	Used late into pregnancy	2.43	(2.32-2.53)	0.97	(0.74-1.27)
2 - Conventional adjusted [c] (population-averaged estimates)	Non-user	1.00		1.00	
	User before pregnancy only	0.90	(0.85-0.94)	0.85	(0.74-0.97)
	Quit during pregnancy	1.28	(1.17-1.40)	1.05	(0.85-1.31)
	Used late into pregnancy	2.37	(2.26-2.49)	1.12	(0.85-1.47)
3 - Unadjusted conditional logistic (within-family estimates)	Non-user	1.00		1.00	
	User before pregnancy only	1.77	(1.54-2.03)	1.42	(0.98-2.04)
	Quit during pregnancy	1.95	(1.51-2.52)	1.16	(0.66-2.02)
	Used late into pregnancy	2.84	(2.26-3.57)	1.30	(0.52-3.26)
4 - Adjusted conditional logistic [c] (within-family estimates)	Non-user	1.00		1.00	
	User before pregnancy only	0.96	(0.83-1.12)	1.00	(0.67-1.50)
	Quit during pregnancy	1.09	(0.83-1.44)	1.25	(0.67-2.33)
	Used late into pregnancy	1.73	(1.36-2.21)	1.98	(0.74-5.28)

[a] Estimates for conventional models produced using a total sample size of 1 017 128 individuals from 676 904 families including 20 949 cases of SGA. Estimates for conditional logistic models produced using a total sample size of 21 147 individuals including 9621 cases of SGA.

[b] Estimates for conventional models produced using a total sample size of 868 933 individuals from 583 286 families including 16 465 cases of SGA. Estimates for conditional logistic models produced using a total sample size of 16 747 individuals including 7616 cases of SGA.

[c] Model adjusted for year of birth, sex, parity, highest parental education, income, parental psychiatric history, maternal country of origin and maternal age at birth.

Supplementary Results

Table B3.5: Sensitivity analysis assuming all those who had younger siblings who were exposed to smoking were also exposed themselves.

			Primary analyses		Sensitivity analyses	
Outcome	Model	Coefficient	O.R.	95% CI	O.R.	95% CI
Intellectual disability [a]	1 - Conventional unadjusted [c]	Population-averaged	1.80	(1.68-1.93)	1.80	(1.68-1.92)
	2 - Conventional adjusted [d]	Population-averaged	1.24	(1.16-1.33)	1.24	(1.16-1.33)
	3 - Within-between unadjusted [c, e]	Within-family	0.91	(0.73-1.14)	0.88	(0.66-1.16)
		Between-family	2.13	(1.70-2.68)	2.16	(1.62-2.87)
	4 - Within-between adjusted [d, e]	Within-family	0.92	(0.74-1.14)	0.88	(0.67-1.16)
		Between-family	1.40	(1.12-1.76)	1.45	(1.08-1.93)
Small for gestational age [b]	1 - Conventional unadjusted	Population-averaged	2.26	(2.18-2.35)	2.22	(2.14-2.30)
	2 - Conventional adjusted [d]	Population-averaged	2.19	(2.11-2.27)	2.12	(2.04-2.20)
	3 - Within-between unadjusted [e]	Within-family	1.68	(1.50-1.89)	2.40	(2.08-2.77)
		Between-family	1.40	(1.23-1.58)	0.92	(0.79-1.07)
	4 - Within-between adjusted [d, e]	Within-family	1.44	(1.27-1.63)	1.38	(1.19-1.61)
		Between-family	1.60	(1.41-1.83)	1.59	(1.35-1.86)

[a] Estimates produced using a total sample size of 1 070 013 individuals from 703 835 families including 6955 cases of ID.

[b] Estimates produced using a total sample size of 1 066 733 individuals from 702 475 families including 22 469 cases of SGA.

[c] Model adjusted for year of birth.

[d] Model adjusted for year of birth, sex, parity, highest parental education, income, parental psychiatric history, maternal country of origin and maternal age at birth.

[e] Model adjusted for family averaged exposure.

Table B3.6: Sensitivity analysis of a restricted cohort of the first two pregnancies study period.

Outcome	Model	Coefficient	Restricted dataset of 1st two children in cohort (Restricted cohort 1) [a]		Discordant sample restricted to those who stop smoking in second pregnancy (Restricted cohort 2) [b]		Discordant sample restricted to those who start smoking in second pregnancy (Restricted cohort 3) [c]	
			O.R.	95% CI	O.R.	95% CI	O.R.	95% CI
Intellectual disability	1 - Conventional unadjusted [d]	Population-averaged	1.77	(1.65-1.90)	1.80	(1.67-1.93)	1.88	(1.75-2.03)
	2 - Conventional adjusted [e]	Population-averaged	1.22	(1.13-1.32)	1.23	(1.14-1.33)	1.27	(1.17-1.37)
	3 - Within-Between unadjusted [d, f]	Within-family	0.86	(0.68-1.10)	0.74	(0.54-1.00)	1.03	(0.70-1.50)
		Between-family	2.22	(1.73-2.86)	2.61	(1.91-3.57)	1.88	(1.28-2.78)
	4 - Within-Between adjusted [e, f]	Within-family	0.87	(0.69-1.11)	0.73	(0.54-1.00)	1.07	(0.74-1.55)
		Between-family	1.46	(1.13-1.88)	1.75	(1.27-2.41)	1.19	(0.82-1.74)
Small for gestational age	1 - Conventional unadjusted	Population-averaged	2.28	(2.20-2.37)	2.37	(2.29-2.47)	2.30	(2.21-2.39)
	2 - Conventional adjusted [e]	Population-averaged	2.18	(2.09-2.27)	2.21	(2.12-2.30)	2.27	(2.18-2.37)
	3 - Within-Between unadjusted [f]	Within-family	1.64	(1.44-1.86)	2.32	(1.99-2.70)	0.86	(0.70-1.06)
		Between-family	1.45	(1.26-1.65)	1.03	(0.87-1.21)	2.78	(2.25-3.43)
	4 - Within-Between adjusted [e, f]	Within-family	1.41	(1.24-1.62)	1.33	(1.12-1.57)	1.59	(1.27-2.00)
		Between-family	1.62	(1.41-1.87)	1.74	(1.46-2.07)	1.44	(1.15-1.81)

[a] Estimates produced using a total sample size of 961 513 individuals for ID analyses and 958 563 individuals for SGA analyses (cases of ID = 6301; SGA = 20 827).

[b] Estimates produced using a total sample size of 951 593 individuals for ID analyses and 948 673 individuals for SGA analyses (cases of ID = 6217; SGA = 20 589).

[c] Estimates produced using a total sample size of 943 759 individuals for ID analyses and 940 868 individuals for SGA analyses (cases of ID = 6170; SGA = 20 360).

[d] Model adjusted for year of birth.

[e] Model adjusted for year of birth, sex, parity, highest parental education, income, parental psychiatric history, maternal country of origin and maternal age at birth.

[f] Model adjusted for family averaged exposure

References

1. Mattsson K, Kallen K, Rignell-Hydbom A, Lindh CH, Jonsson BA, Gustafsson P, et al. Cotinine Validation of Self-Reported Smoking During Pregnancy in the Swedish Medical Birth Register. Nicotine Tob Res. 2016;18(1):79-83.
2. The National Board of Health and Welfare. The Swedish Medical Birth Register - A Summary of Content and Quality 2003 [Available from: https://www.socialstyrelsen.se/publikationer2003/2003-112-3.
3. Sandin S, Hultman C, Reichenberg A. Autism in children born after in vitro fertilization--reply. JAMA. 2013;310(19):2101.
4. Sandin S, Lichtenstein P, Kuja-Halkola R, Larsson H, Hultman CM, Reichenberg A. The familial risk of autism. JAMA. 2014;311(17):1770-7.
5. Ludvigsson JF, Andersson E, Ekbom A, Feychting M, Kim JL, Reuterwall C, et al. External review and validation of the Swedish national inpatient register. BMC Public Health. 2011;11:450.
6. Idring S, Rai D, Dal H, Dalman C, Sturm H, Zander E, et al. Autism spectrum disorders in the Stockholm Youth Cohort: design, prevalence and validity. PLoS One. 2012;7(7):e41280.
7. Ruck C, Larsson KJ, Lind K, Perez-Vigil A, Isomura K, Sariaslan A, et al. Validity and reliability of chronic tic disorder and obsessive-compulsive disorder diagnoses in the Swedish National Patient Register. BMJ Open. 2015;5(6):e007520.
8. Statistics Sweden. Longitudinal integration database for health insurance and labour market studies (LISA by Swedish acronym). English; 2012.
9. Ekbom A. The Swedish Multi-generation Register. Methods Mol Biol. 2011;675:215-20.
10. Frisell T, Oberg S, Kuja-Halkola R, Sjolander A. Sibling comparison designs: bias from non-shared confounders and measurement error. Epidemiology. 2012;23(5):713-20.
11. Keyes KM, Smith GD, Susser E. On Sibling Designs. Epidemiology. 2013;24(3):473-4.
12. Sjolander A, Frisell T, Kuja-Halkola R, Oberg S, Zetterqvist J. Carryover Effects in Sibling Comparison Designs. Epidemiology. 2016;27(6):852-8.
